# Matrix metalloproteinase-guided targeting of vesicular stomatitis virus to tumors following systemic administration

**DOI:** 10.1038/s41392-026-02888-x

**Published:** 2026-10-20

**Authors:** Eileen Victoria Theune, William C. Theune, Elham Ahmadi, Conner C. Chiari, Neha Singh, Rory Geyer, Jason Tonne, Muriel Metko, Olamide T. Olaoba, Isabella China Wang, Dylan Dai, Jianbin Ruan, Robert Clark, Guangfu Li, Penghua Wang, Evan R. Jellison, Anthony T. Vella, Richard Vile, Kepeng Wang

**Affiliations:** 1 Department of Immunology, University of Connecticut School of Medicine, UConn Health, Farmington, CT USA; 2 Department of Molecular Medicine, Mayo Clinic, Rochester, MN USA; 3 Department of Surgery, University of Connecticut School of Medicine, UConn Health, Farmington, CT USA; 4 Johns Hopkins University, Baltimore, MD USA; 5 University of Connecticut, Storrs, CT USA; 6 Department of Immunology, Mayo Clinic, Rochester, MN USA; 7 Comprehensive Cancer Centre, School of Cancer & Pharmaceutical Sciences, School of Immunology and Microbial Sciences, King’s College London, London, UK

**Keywords:** Gene therapy, Drug development, Immunotherapy, Gene delivery

## Abstract

The lack of tumor-specific targeting by oncolytic viruses (OVs) restricts their therapeutic potential. Here, we report a “cap-linker” strategy to modify the vesicular stomatitis virus (VSV) glycoprotein (G protein, VSV-G^IN^). The modified VSV (Pro-VSV^IN^) loses its ability to infect cells until present in the tumor microenvironment, where the linker is cleaved by tumor-enriched matrix metalloproteinases (MMPs). When Pro-VSV^IN^ is armed with a single-chain IL-12 (Pro-VSV^IN^-IL-12) and systemically injected into MC38 tumor-bearing mice, Pro-VSV^IN^-IL-12 eradicates established tumors while avoiding systemic toxicity, and elicits long-term anti-tumor immune memory. Systemic administration of Pro-VSV^IN^-IL-12 also markedly inhibits the growth of metastatic cancers in the lung. The efficacy of Pro-VSV^IN^-IL-12 was further improved when combined with PD-1 checkpoint blockade. Compared to WT VSV^IN^ that carries IL-12, intravenously injected Pro-VSV^IN^-IL-12 leads to strong Th1 immunity, near abolishment of regulatory T cells, and potent CD8^+^ T cell activation and clonal expansion in tumors. This preclinical work demonstrates a new tumor-specific targeting strategy of VSV for enhanced immunotherapy safety with superior efficacy.

## Introduction

Oncolytic viruses (OVs) are emerging immunotherapies under active clinical investigation.^1^ These agents, which can be used in their wild type or engineered forms, preferentially infect, replicate in, and lyse cancer cells, exposing tumor antigens to the host immune system to help facilitate an anti-cancer immune response. Currently, four OVs, and one non-replicating OV, are approved for use clinically, each of which require intratumoral administration.^2–6^ Additional agents under preclinical investigation are engineered to harbor immune-enhancing and costimulatory payloads, or even suicide cassettes to bolster anti-tumor immunity.^7–9^ In many cases, clinical applications of these agents are limited by their current requirement for local injection. This precludes tumors that are not surgically accessible, or are metastatic. For OVs that are injected systemically, the lack of a “tumor-specific” viral delivery platform limits the choice of immune activating payloads to avoid severe toxicities.^8–10^ Further clinical development of novel viral vectors that have refined specificity for targeting tumors is needed.

Vesicular stomatitis virus (VSV) belongs to the *Rhabdoviridae* family of viruses, having a single-stranded, negative-sense RNA genome of around 11 kb.^11,12^ VSV mainly infects livestock and causes blister-like lesions in these animals.^11,12^ While VSV infection in humans is rare, it can cause flu-like symptoms.^11^ VSV replicates strictly in the host cell’s cytoplasm, does not enter the nucleus, and does not interact with host DNA. This feature, combined with a lack of a DNA phase for VSV, makes it safer compared to DNA viruses (such as Adenovirus) due to the absence of potential insertional mutagenesis to the host.^11,12^ Owing to its low prevalence (and thus less prior existing immunity against the vector) and mild symptoms in humans, VSV has been studied as a potential vector for cancer immunotherapy.^11,13^ For instance, the human IFN-β transgene has been cloned into the VSV backbone to further enhance its viral safety profile.^14–16^ Others incorporated interleukin-12 (IL-12) into VSV, and showed that intratumoral injection of these viruses control tumor burden.^17,18^ VSV harbors its G protein (VSV-G) on the surface of the membrane, which binds to the low-density lipoprotein receptor (LDL-R) on host cells to begin the cell entry process. A recent publication further narrows down the interaction between VSV-G to the CR-2 and CR-3 domains of the LDL-R.^19^ Due to the wide expression pattern of the LDL-R across different tissues and cell types, the VSV-G protein was used to replace the spike protein of HIV to generate lentivirus that is now commonly used in laboratories.^20^ However, the broad-tropism of the VSV-G protein also determines the lack of tumor specificity of VSV, limiting its route of administration to intratumoral injection when potent payloads, such as IL-12, are incorporated.

Although current immunotherapies have demonstrated clinical success, response rates to these therapies vary dramatically between cancer type, and person.^21–24^ For instance, colorectal cancer (CRC) is the third leading cause of cancer-related deaths in the United States,^25^ and only a small fraction (15%) of CRC that harbor microsatellite instability (MSI) mutations are responsive to immune checkpoint blockade (ICB) therapies such as anti-PD-1.^26^ Additionally, for advanced CRCs that are microsatellite stable (MSS), immunotherapeutic options are limited and there is a critical need for better treatments. The success of ICB is limited by factors including tumor immunoediting, mutational burden, and the presence and activation state of infiltrating lymphocytes.^27^ To overcome therapeutic limitations observed with ICB, OVs are used to inflame the tumor microenvironment (TME) and promote T cell responses directed against tumor antigens, as well as increase the expression of checkpoint molecules on immune and tumor cells.^28–34^ Currently, various clinical trials (NCT03017820, NCT03647163, NCT03120624, NCT06581406, NCT04735978, NCT03767348, NCT04725331) are actively investigating the efficacy of these therapeutic combinations, as preclinical trials have pointed towards a synergy from dual treatments.^29–32^ However, the efficacy of the combination therapy is heavily dependent on several factors not limited to the type of tumor, strain of OV, transgene payload, as well as the dosing scheme, underscoring the importance of understanding how each unique OV construct impacts the efficacy of the combination therapy.

Here, we demonstrate the development of a cancer-specific oncolytic VSV by engineering the endogenous G protein with a “cap-linker” strategy. This modification allows for the safe systemic delivery of potent, immune-stimulating IL-12 directly to the site of the tumor resulting in tumor eradication. We also interrogated the efficacy of the engineered virus in treating metastatic cancers and its ability to mount long-term immune memory. Importantly, we combined tumor-targeted delivery of IL-12 with ICB for enhanced efficacy against established tumors. These efforts pave a new way of applying our novel Pro-VSV technology to safely deliver potent immune activating payloads to human cancers that are not surgically reachable. Further, we generalized our study to include G proteins from two serotypes of VSV and the Maraba virus, to show that the “cap-linker” strategy may be generally applicable for targeted tumor delivery of oncolytic viruses.

## Results

### Rational design of a cancer-specific viral platform using lentivirus

VSV-G binds to LDL-R on target cells, resulting in broad tropism in humans and experimental animals.^19,35^ Specifically, VSV’s clinical use is limited by its potential for neurotoxicity and infectivity of the heart and kidney, which is observed in mice and non-human primates, as well as hepatotoxicity.^36–38^ Lack of tumor specificity by VSV thus limits the choice of payload that the virus may carry, with immune-related adverse events as a concern if potent immune activating payloads (such as IL-12) are used.

**Fig. 1:**
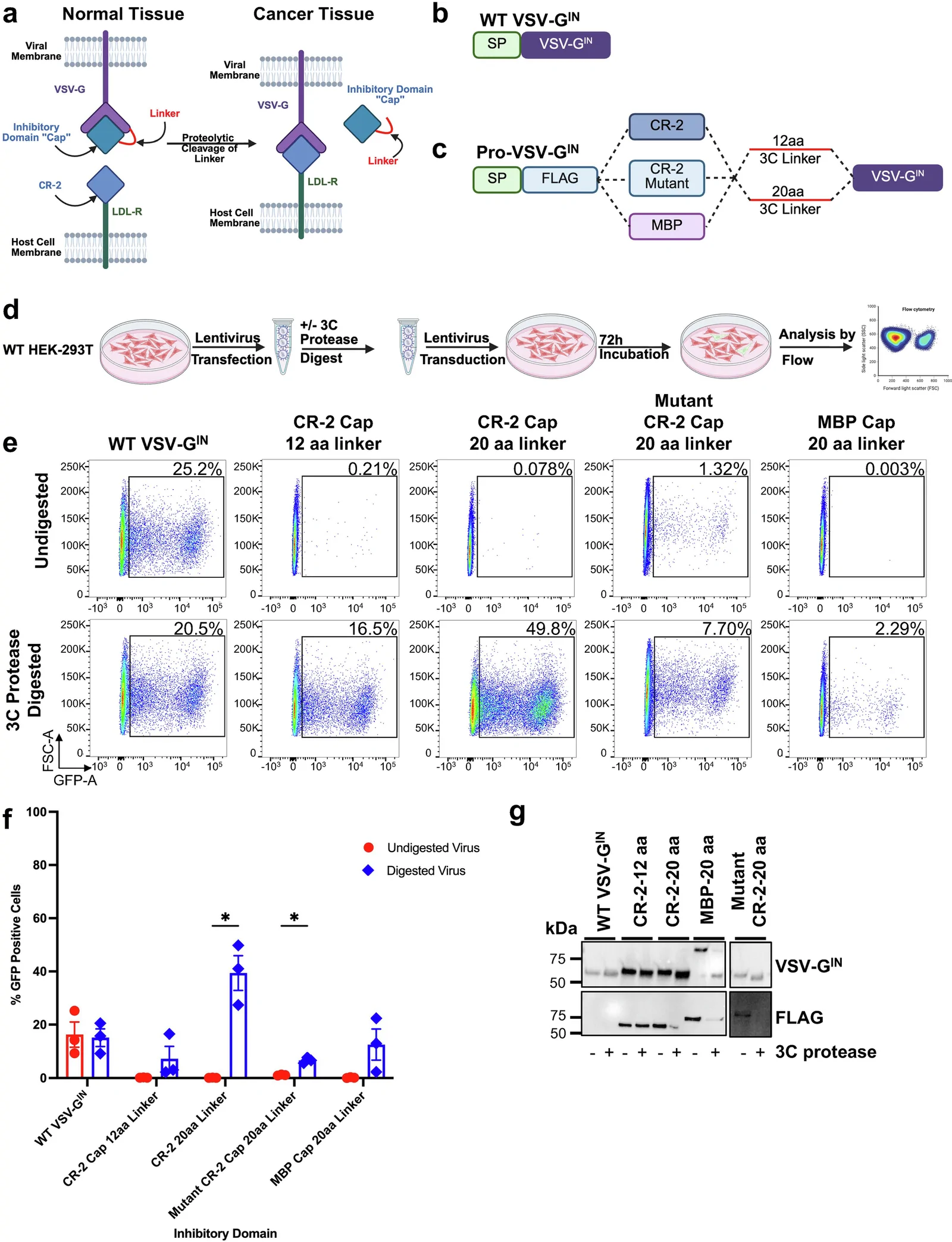
Cap-linker strategy to modify of VSV-G^IN^. **a** Illustration of the ‘cap-linker’ strategy for the development of a cancer-specific oncolytic virus. An inhibitory domain of the VSV-G protein (the “cap”) is engineered to the N’-terminal of VSV-G, spaced by a protease-cleavable linker. Loss of the cap occurs upon viral entry into cancer tissue where the overexpression of proteases allows for the facile cleavage of the amino acid linker sequence that tethers the cap to VSV-G, restoring viral infectivity. **b**, **c** Illustration of the domain structure for WT VSV-G^IN^ (**b**) and Pro-VSV-G^IN^ (**c**), the latter contains an N’-terminal addition of a FLAG tag with different inhibitory domains linked to mature VSV-G^IN^ by either a 12 or 20 amino acid 3C protease cleavable linker sequence. The mutant CR-2 domain carries W66A, D69A, Q71A, D73A mutations. SP = signal peptide. MBP = Mannose binding protein. **d** Schematic for the optimization of the inhibitory domain and linker length for Pro-VSV-G^IN^ through the generation of GFP-expressing lentiviruses pseudotyped with WT or Pro-VSV-G^IN^ in HEK-293T cells, with or without proteolytic digestion using 3C protease prior to transduction of HEK-293T cells and flow cytometry analysis. **e**, **f** GFP-expressing lentiviruses pseudotyped with WT VSV-G^IN^ or Pro-VSV-G^IN^ with different inhibitory domains and linker lengths were produced by HEK-293T cells and used to transduce HEK-293T cells, followed by flow cytometry analysis. **e** Representative flow plot of GFP signal in cells following viral infection. In the upper panels of (**e**), viruses were added onto cells in their native form. In the lower panel of (**e**), viruses were digested with 3C protease (20 U/ml) for 3 h on ice prior to cell transduction. Quantification of GFP^+^ cells are shown in (**f**). **g** Western blot analysis of VSV-G^IN^ and FLAG expression before and after 3C protease digest of WT and Pro-VSV-G^IN^ lentiviruses to confirm linker cleavage and removal of inhibitory cap

To improve the cancer-specific targeting of systemically injected VSV, we engineered VSV-G^IN^ (Indiana serotype) to limit its ability to bind its cognate receptor unless present in the tumor microenvironment (TME). We developed a ‘cap-linker’ cancer-specific targeting system where the ‘cap’ acts as an inhibitory domain to VSV-G^IN^, but is released when the linker connecting the cap to VSV-G^IN^ is cleaved by proteases in the TME. This results in the rescue of VSV-G^IN^’s ability to bind to the LDL-R and guide viral entry into tumor cells (Fig. 1a).

We first utilized the lentiviral platform to determine the optimal cap and linker length to mediate viral inhibition. We used WT VSV-G^IN^ (Fig. 1b) and Pro-VSV-G,^IN^ which contains the cap-linker modification (Fig. 1c). For the Pro-VSV-G^IN^ constructs, we selected the cysteine-rich 2 (CR-2) domain (~ 4.5 kDa) from the LDL-R as an inhibitory domain (cap), and cloned it to the N’-terminus of the mature VSV-G^IN^ after the endogenous signal peptide and connected it to VSV-G^IN^ by a 12- or 20-amino acid (aa) protease cleavable linker (Fig. 1c).^19^ We also generated a CR-2 cap which harbors four different point mutations at the amino acids that have been shown to be critical for mediating VSV-G’s binding to the CR-2 domain (Fig. 1c).^19^ Additionally, we investigated the use of a mannose binding protein (MBP) (~43 kDa) cap, in place of the CR-2 domain, and connected it to VSV-G^IN^ with a 20 aa linker (Fig. 1c). Finally, we used a 3C protease cleavable linker to avoid inadvertent activation of Pro-VSV-G^IN^ pseudotyped viruses by proteases endogenous to the HEK-293T cells used for viral production and screening.^39^

To probe the infectious activity of Pro-VSV-G^IN^ pseudotyped viruses, we transduced HEK-293T cells with WT VSV-G^IN^ or Pro-VSV-G^IN^ pseudotyped, GFP-encoding lentiviruses prior to or directly following 3C protease digest and analyzed the number of infected cells by GFP signal (Fig. 1d). Compared to lentivirus harboring WT VSV-G^IN^, the infectious activity of Pro-VSV-G^IN^ pseudotyped viruses, prior to 3C protease digest, was completely abolished with all constructs, except Pro-VSV-G^IN^ harboring the mutated CR-2 cap (Fig. 1e, upper panels). We next treated viruses with 3C protease and examined the degree of restoration of lentivirus infectivity (Fig. 1e, lower panels). While only a marginal rescue of the viral titer was observed with the removal of the MBP or mutant CR-2 caps, the removal of the CR-2 cap resulted in complete restoration of lentivirus infection, with the 20 aa linker length proving to be robust beyond that of the WT VSV-G^IN^ control (Fig. 1e–g). The Pro-VSV-G^IN^ pseudotyped lentivirus with a CR-2 cap and 20 aa linker showed a near 700-fold activation compared to its undigested counterpart, demonstrating the versatility of the cap-linker strategy in VSV-G inhibition and protease-dependent activation. The higher signal of the Pro-VSV-G^IN^ pseudotyped lentivirus (CR-2 cap, 20 aa linker) may be attributed to the inability of the virus to infect host cells during viral production, resulting in enhanced viral accumulation in the supernatant.

### Optimization of a cancer-specific virus using viral spike protein cognate binding domains

With the success of the CR-2 domain as an effective cap in our engineered Pro-VSV-G^IN^ (Fig. 1), we next compared the efficacy of the additional six CR domains of the LDL-R for their ability to inhibit VSV-G^IN^ function. We included the New Jersey (NJ) serotype of VSV-G (VSV-G^NJ^), which is more pathogenic than the Indiana serotype,^40,41^ and investigated the Maraba virus glycoprotein which uses the LDL-R for cell entry and is more resistant to serum neutralization.^42^

**Fig. 2:**
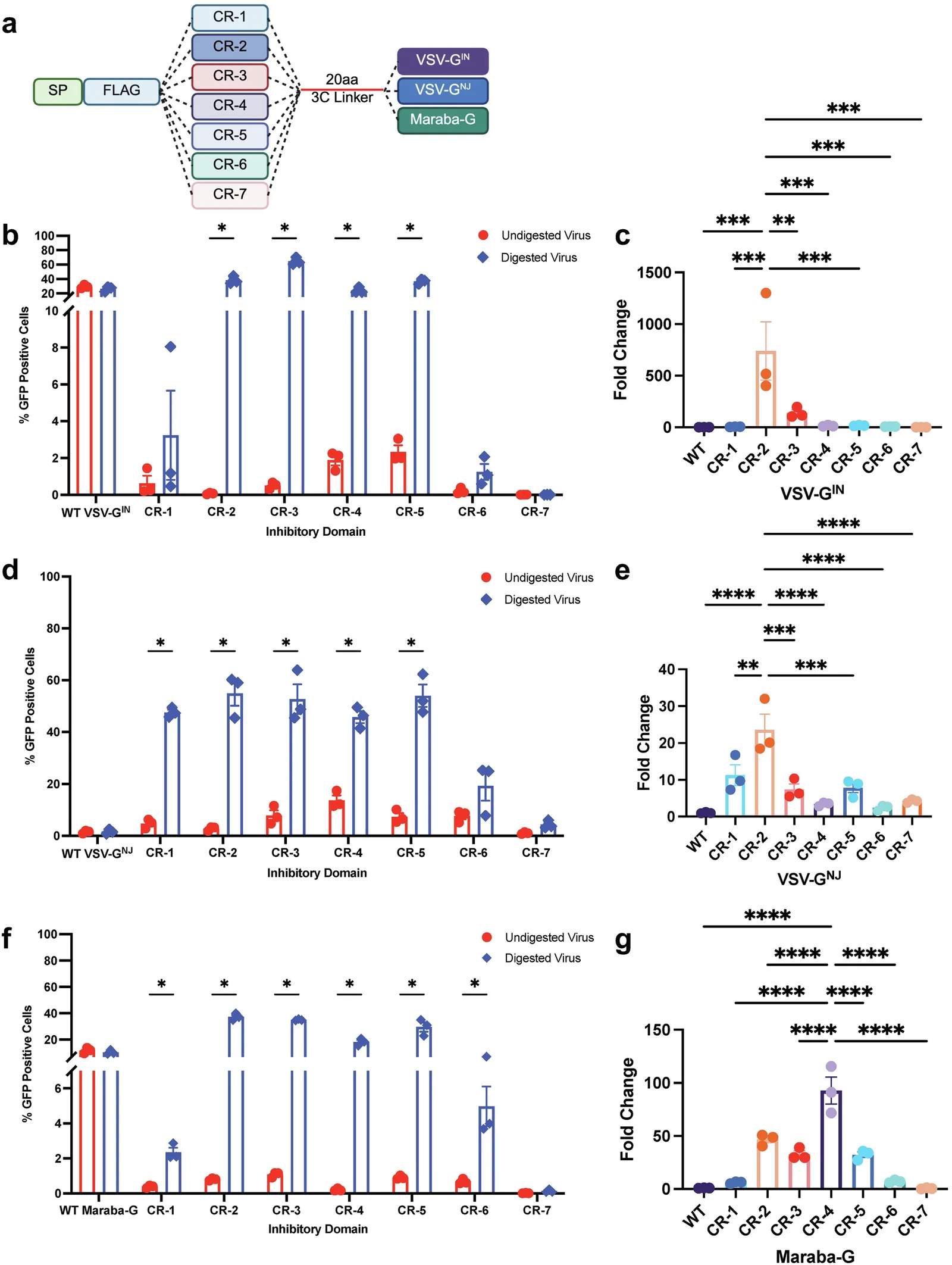
Optimization of the cap-linker strategy for VSV-G^IN^, VSV-G^NJ^, and Maraba-G. **a** Schematic of the investigation testing the use of LDL-R cysteine rich (CR) domains 1–7 as inhibitory domains for Pro-VSV-G^IN^, Pro-VSV-G^NJ^, or Pro-Maraba-G constructs. **b**, **d**, **f** Flow cytometry analysis of HEK-293T cells infected with GFP-expressing lentiviruses pseudotyped with (**b**) WT VSV-G^IN^ and Pro-VSV-G^IN^, (**d**) WT VSV-G^NJ^ and Pro-VSV-G^NJ^, and (**f**) WT Maraba-G and Pro-Maraba-G. **c**, **e**, **g** Fold of activation as calculated by analyzing GFP^+^ HEK-293T cells 72 h post infection, and dividing the viral titer of each strain with 3C protease digested vs undigested viruses for (**c**) VSV-G^IN^, (**e**) VSV-G^NJ^ (**g**) Maraba-G, *n* = 3. All data represent consistent findings in three independent experiments

We covalently linked the CR-1 through CR-7 domains of the LDL-R to the N’-terminus of VSV-G^IN^, VSV-G^NJ^, or Maraba-G, using the 20 aa 3 C protease cleavable linker (Fig. 2a). We transduced HEK-293T cells and quantified viral titer by assessing GFP^+^ cells 3 days post infection. The CR-2 domain provided the greatest fold of activation for Pro-VSV-G^IN^ and Pro-VSV-G^NJ^ (Fig. 2b–e; supplementary Figs. 1 and 2); however, the higher basal level of activity of Pro-VSV-G^NJ^ in its native form reduced the extent of activation of the virus compared to the Indiana serotype (Fig. 2c, e). Interestingly, unlike both VSV-G serotypes, Pro-Maraba-G pseudotyped lentivirus demonstrated the greatest fold of activation when utilizing the CR-4 domain as a cap, although to a much lesser extent than Pro-VSV-G^IN^ (Fig. 2f, g; supplementary Fig. 3).

Thus, the CR-2 domain as an inhibitory cap connected to VSV-G^IN^ with a 20 aa cleavable linker provides the cleanest signal-to-noise ratio across all investigated modified glycoproteins.

### Engineering of Pro-VSV-G^IN^ pseudotyped lentivirus for tumor-specific protease activation

**Fig. 3:**
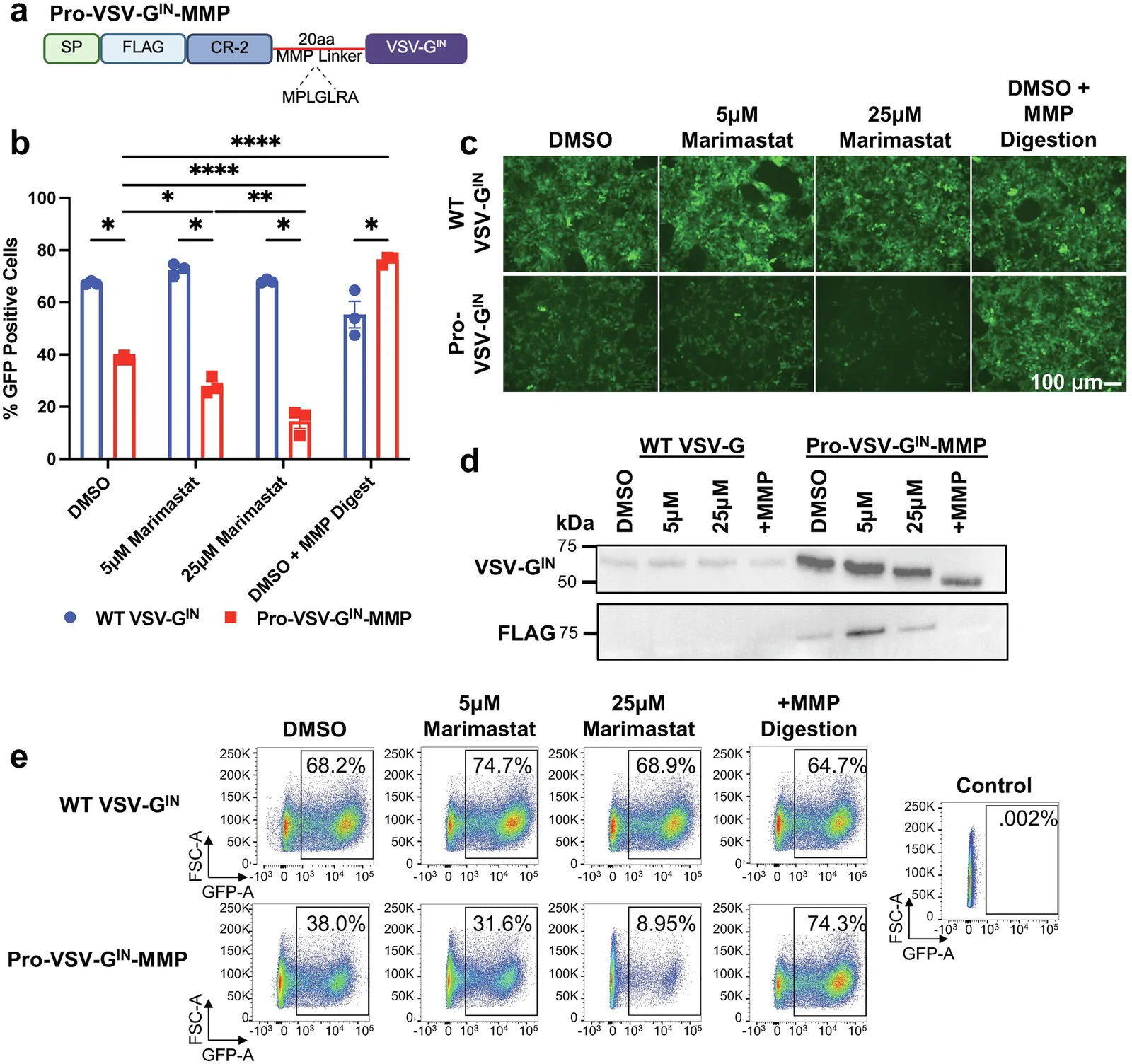
MMP-dependent activation of Pro-VSV-G^IN^-MMP. **a** Illustration of the design for Pro-VSV-G^IN^-MMP which harbors a CR-2 inhibitory domain and a 20 aa flexible, pan-MMP cleavable linker fused to the N’-terminus of VSV-G^IN^. **b**–**e** WT VSV-G^IN^ and Pro-VSV-G^IN^-MMP containing plasmids were co-transfected with lentiviral helper plasmids to HEK-293T cells to generate lentiviruses coated with WT or Pro-VSV-G^IN^-MMP spike proteins, respectively. Cell supernatant containing lentivirus was used to transduce HEK-293T cells in 35 mm dishes. During viral production and transduction, indicated concentrations of broad-spectrum MMP inhibitor, marimastat, were added to cell culture medium. Additionally, once generated, Pro-VSV-G^IN^-MMP coated lentivirus was digested with 0.3 U/ml of MMP-7 for 1 h prior to infecting HEK-293T cells and analyzed 72 h following viral transduction by **b** Flow cytometry, *n* = 3, **c** fluorescent cell imaging, and **d** western blotting using viral supernatants for the presence of VSV-G^IN^ and FLAG across sample conditions. **e** Representative flow plots and percentage of virally infected, GFP^+^ HEK-293T cells

Among different proteases, the matrix metalloproteinase (MMP) family has been shown to be upregulated across different human cancers.^39,43–47^ Additionally, MMP activity is known to promote tumor development by aiding angiogenesis and metastasis.^48^ The abundance of MMPs across multiple tumor types makes them ideal for use as an activating signal for the liberation of Pro-VSV-G^IN^.^39,43–47^ Therefore, we engineered our 20-aa linker to contain a universal MMP cleavage site (Pro-VSV-G^IN^-MMP, Fig. 3a).

The incorporation of an MMP-cleavable linker resulted in a viral activity that was only marginally lower than the WT virus (Fig. 3b–e), which was due to HEK-293T cells producing MMPs. Addition of marimastat, a broad-spectrum MMP inhibitor that blocks MMPs 1, 2, 3, 7, 8, 9, and 14, resulted in a dose-dependent decrease in the viral titer of Pro-VSV-G^IN^-MMP lentivirus (Fig. 3b–e).^49^ Pro-VSV-G^IN^-MMP lentivirus pre-digested with recombinant MMP-7 prior to infection, resulted in a significant increase in the percent of virally infected cells compared to the marimastat-treated counterparts (Fig. 3b–e). Taken together, these data demonstrate the MMP-dependent activity of Pro-VSV-G^IN^-MMP in guiding viral entry into the cell.

### CRISPR knockout of MMPs in HEK-293T cells allows for efficient production of Pro-VSV-G^IN^ pseudotyped lentivirus in its native form

We suspected that the remnant activity of Pro-VSV-G^IN^-MMP pseudotyped lentivirus was due to the expression of MMPs in HEK-293T cells not targeted by marimastat. RT-qPCR analysis of all MMP mRNAs show that MMPs 2, 11, 15, 16, and 17 are expressed at relatively high levels in HEK-293T cells (supplementary Fig. 4a), which prompted us to engineer HEK-293T cells using CRISPR/Cas9 technology to knockout all five MMPs (supplementary Fig. 4b, MMP-KO HEK-293T cells). Successful knockout of these genes was confirmed by Sanger sequencing of genomic DNA from single MMP-KO HEK-293T cell clones, and we selected the best clone C2-11 for subsequent study (supplementary Fig. 4b, c). We next performed a functional test to investigate the ability of our MMP-KO HEK-293 T (clone C2-11) to produce lentivirus (supplementary Fig. 4d) and showed that the MMP-KO HEK-293T cells were as efficient as WT HEK-293T cells in producing lentiviruses (supplementary Fig. 4e). The activity of Pro-VSV-G^IN^-MMP lentivirus produced in MMP-KO HEK-293T cells is abolished when used to infect MMP-KO HEK-293T cells and is completely rescued when recombinant MMP-7 is used to pre-digest the virus prior to infection of these cells (supplementary Fig. 4f, g). Thus, the MMP-KO HEK-293T cell line serves as an ideal platform to produce MMP-dependent viruses by preventing premature MMP cleavage on the viral particles.

### Systemic injection of Pro-VSV-G^IN^-MMP pseudotyped vesicular stomatitis virus exhibits a tumor-restricted tropism

**Fig. 4:**
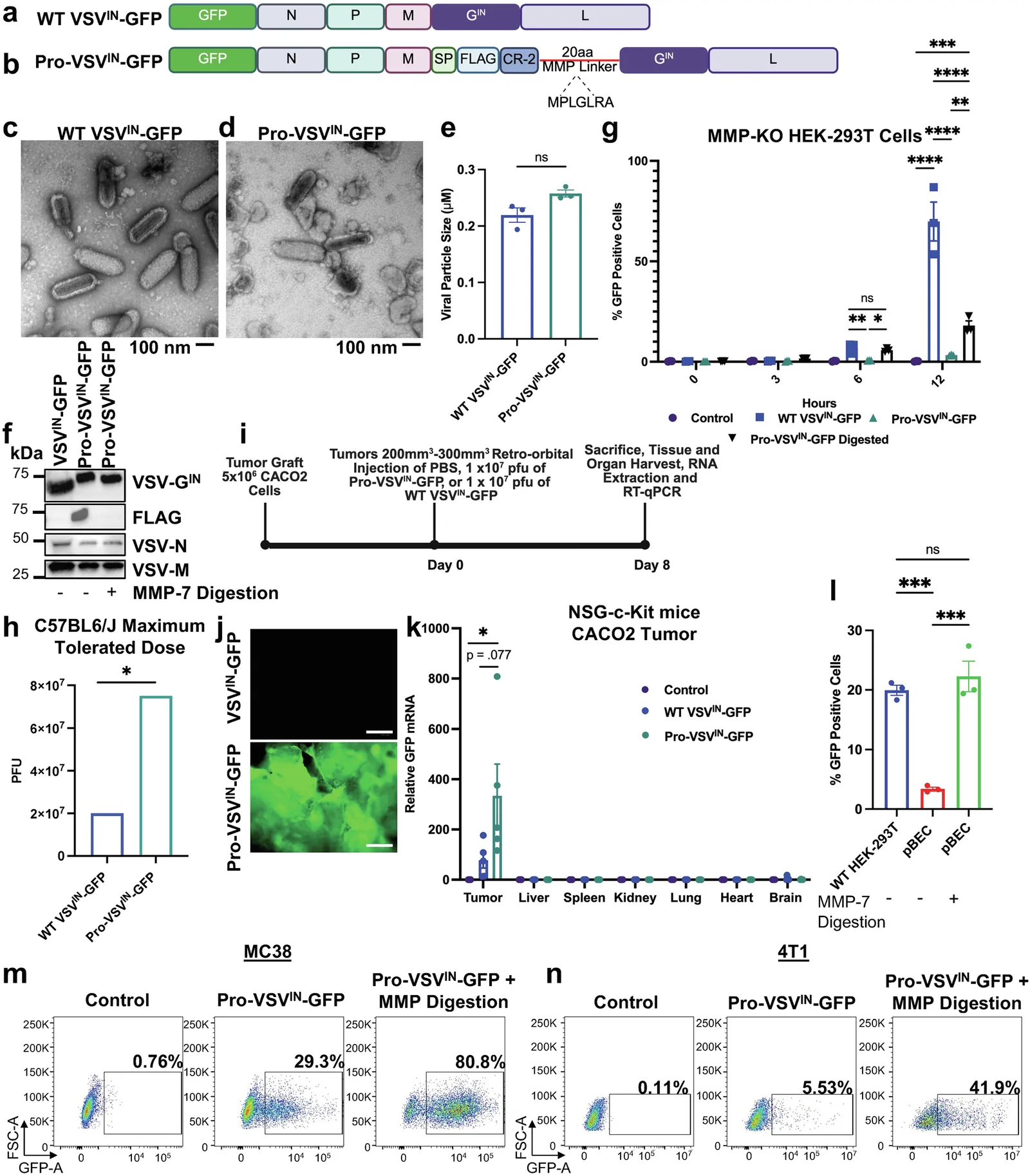
Engineering VSV with Pro-VSV-G^IN^-MMP enhances safety and tumor specificity. **a** Schematic for the design of EGFP transgene expressing WT VSV^IN^ (WT VSV^IN^-GFP). **b** Schematic for the design of an EGFP transgene expressing Pro-VSV^IN^ (Pro-VSV^IN^-GFP) which possesses a Pro-VSV-G^IN^-MMP spike protein harboring an N’-terminal FLAG tag, and CR-2 inhibitory domain connected to VSV-G^IN^ by a 20 aa flexible, MMP cleavable linker (MPLGLRA). **c**, **d** TEM at a 50,000x magnification of WT VSV^IN^-GFP (**c**) and Pro-VSV^IN^-GFP (**d**). **e** Quantification of TEM viral particle size analysis of WT VSV^IN^-GFP and Pro-VSV^IN^-GFP, *n* = 3. **f** Western blot detection of viral proteins (VSV-G, VSV-N, and VSV-M), as well as Pro-VSV^IN^-GFP FLAG tag from purified viruses. Recombinant MMP-7 protease was used to digest Pro-VSV^IN^-GFP, to remove the FLAG and CR-2 inhibitory domain prior to Western blot analysis (third lane), resulting in a downward shift of the VSV-G^IN^ detection band. **g** Quantification by flow cytometry of the number of GFP^+^ cells at 0, 3, 6, and 12 h following viral transduction of C2-11 MMP-KO HEK-293T cells with VSV^IN^-GFP, Pro-VSV^IN^-GFP, and MMP-7 protease pre-digested Pro-VSV^IN^-GFP viruses at an MOI of 0.1, *n* = 3. Uninfected cells were used as controls. **h** Observed maximum tolerated dose (MTD) for WT VSV^IN^-GFP and Pro-VSV^IN^-GFP injected i.v. into C57BL/6J mice. Mice were observed for up to 2 months post injection. The MTD was determined to be the highest dose with no observed treatment related paralysis or death, *n* = 5. **i**–**k** NSG-c-Kit mice were grafted with 5 × 10^6^ CACO2 human colon cancer cells subcutaneously on one flank. One month later, when tumor sizes reached 200 to 300 mm^3^, mice received i.v. injection of 1 × 10^7^ pfu of either WT VSV^IN^-GFP or Pro-VSV^IN^-GFP (Day 0). Mice were sacrificed 8 days post treatment, followed by collection of tumor tissue and indicated organs which were examined for (**j**) green fluorescence by microscopy, and (**k**) the level of GFP mRNA by RT-qPCR, *n* = 5. Scale bars = 100 nm for (**c**) and (**d**), and 100 μm for (**j**). **l** Primary human brain microvascular endothelial cells (pBECs) were transduced with Pro-VSV^IN^-GFP, or Pro-VSV^IN^-GFP digested with 0.3 U/ml of MMP-7 for 1 h prior to infection at an MOI of 0.25. WT HEK-293T cells were also transduced with Pro-VSV^IN^-GFP at an MOI of 0.25. Cells were analyzed by flow cytometry for GFP expression at 8 h post infection, *n* = 3. **m**, **n** Representative flow cytometry analysis of MC38 (**m**) or 4T1 (**n**) transduced with Pro-VSV^IN^-GFP, or Pro-VSV^IN^-GFP digested with 0.3 U/ml of MMP-7 for 1 h prior to infection at an MOI of 0.25. Cells were analyzed by flow cytometry for GFP expression 8 h later. Uninfected cells were used as controls

Given the many attractive qualities of VSV, we replaced the VSV-G^IN^ spike protein on an EGFP-expressing VSV^IN^ (VSV^IN^-GFP) with Pro-VSV-G^IN^-MMP and designated this recombinant VSV as Pro-VSV^IN^-GFP (Fig. 4a, b). As anticipated, the structures of WT VSV^IN^-GFP and Pro-VSV^IN^-GFP under transmission electron microscopy (TEM) resembles the classic bullet-shape for VSV, confirming that the genetic modification made to Pro-VSV^IN^-GFP did not compromise the overall capsid structure of the virus (Fig. 4c–e). We next probed the conservation of the MMP-dependent activation of Pro-VSV^IN^-GFP and confirmed the ability of the CR-2 cap to be removed (Fig. 4f). Importantly, when added to MMP-KO HEK-293T cells, we found that Pro-VSV^IN^-GFP showed a marked decrease in viral spreading, as observed by flow cytometry at timepoints across 12 h, compared to cells transduced with WT VSV^IN^-GFP (Fig. 4g). However, MMP-7 pre-digestion of Pro-VSV^IN^-GFP restored the ability of the virus to infect cells at 6 h, although viral spreading was slower at 12 h compared to WT VSV^IN^-GFP because the newly produced Pro-VSV^IN^-GFP was again capped (Fig. 4g).

We examined the safety profile of the Pro-VSV^IN^-GFP compared to WT VSV^IN^-GFP. Using non-tumor bearing, immune competent C57BL/6J mice, we probed the safety profile by dose escalation to determine the maximum tolerated dose (MTD) of each virus. Pro-VSV^IN^-GFP proved to have a nearly 4-times higher MTD than WT VSV^IN^-GFP (Fig. 4h).

We then performed a biodistribution analysis over eight days using CACO2 human colon tumor bearing NSG-c-KIT mice intravenously (i.v.) injected with 1 × 10^7^ pfu of Pro-VSV^IN^-GFP. Pro-VSV^IN^-GFP preferentially expanded in the tumor environment (Fig. 4i–k, supplementary Fig. 5a). Compared to all normal tissues examined, 500 times the amount of GFP RNA was detected in the tumor of mice treated with Pro-VSV^IN^-GFP (Fig. 4i–k). The level of Pro-VSV^IN^-GFP quickly elevated in the tumor through the 8-day period, suggesting robust replication of the virus in the tumor environment (supplementary Fig. 5a). Additionally, when compared to systemic injection of WT VSV^IN^-GFP into CACO2 bearing NSG-c-Kit mice, Pro-VSV^IN^-GFP showed much greater accumulation in the tumor (Fig. 4j, k). VSV infects liver cells,^50^ and H/E staining of liver showed that WT VSV^IN^-GFP, but not Pro-VSV^IN^-GFP, caused marked damage (supplementary Fig. 5b). Pro-VSV^IN^-GFP readily infects HEK-293T cells, but not primary human brain microvascular endothelial cells (pBEC) (Fig. 4l). Pre-digestion of Pro-VSV^IN^-GFP with MMP rescued its defective infection in the non-cancerous endothelial cells (Fig. 4l). In addition, Pro-VSV^IN^-GFP also infects two mouse cancer cell lines, MC38 and 4T1, and its viral activity is further enhanced when the virus is pre-digested with MMP (Fig. 4m, n). Collectively, our data demonstrate that Pro-VSV^IN^-GFP is tumor-specific and possess an excellent safety profile.

### Systemic administration of Pro-VSV^IN^ expressing a single-chain interleukin-12 transgene eradicates MC38 murine colorectal cancer tumors

Interleukin (IL)-12 (IL-12) possesses potent anti-tumor activity,^51,52^ but systemic delivery of recombinant IL-12 resulted in severe toxicity and even death in clinical trials.^53–55^ To avoid the toxicity of IL-12, but harness its anti-cancer properties, OVs have been investigated to improve the safety profile of the cytokine and express the cytokine locally in the tumor.^17,56–72^ Given the improved safety profile and enhanced tumor-specificity observed with Pro-VSV^IN^-GFP compared to that of WT VSV^IN^-GFP (Fig. 4), we hypothesized that upon systemic injection, Pro-VSV^IN^ engineered to express a single-chain (sc) IL-12 (sc-IL-12) would be safe and effective.

**Fig. 5:**
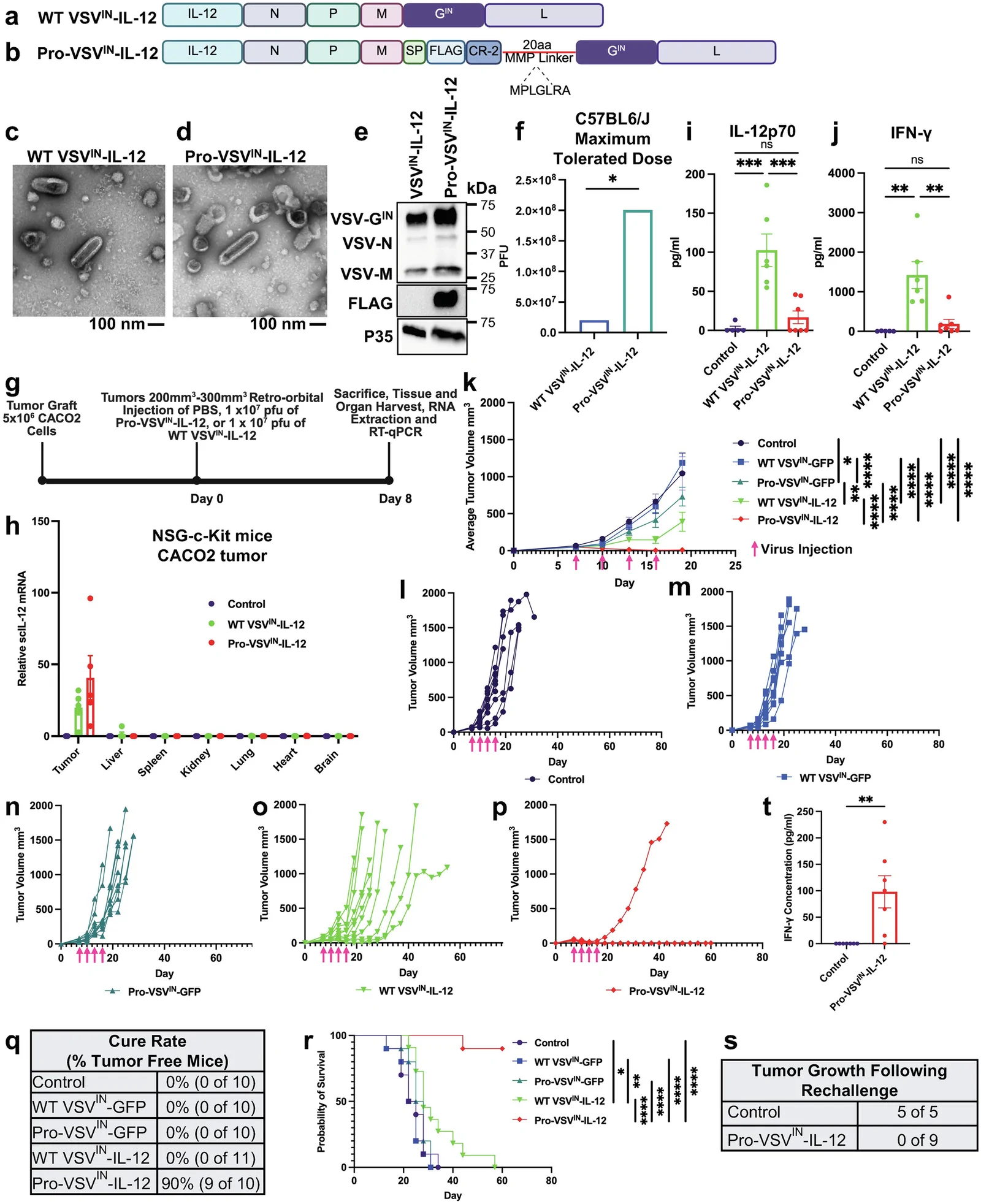
Systemic injection of Pro-VSV^IN^-IL-12 eradicates tumors. **a** Schematic for scIL-12 expressing WT VSV^IN^ (WT VSV^IN^-IL-12). **b** Schematic for the design of scIL-12 expressing Pro-VSV^IN^ (Pro-VSV^IN^-IL-12). **c**, **d** TEM at a 50,000x magnification of (**c**) WT VSV^IN^-IL-12 and (**d**) Pro-VSV^IN^-IL-12. **e** Western blot detection of viral proteins (VSV-G, VSV-N, and VSV-M), and Pro-VSV^IN^-GFP FLAG tag from purified viruses. Detection of scIL-12 performed by harvesting viral supernatant from infected BHK-21 cells. **f** Increasing doses of WT VSV^IN^-IL-12 and Pro-VSV^IN^-IL-12 were injected i.v. into C57BL/6J mice. Mice were observed for up to 15 days post injection. MTDs were determined to be the dose where there was no treatment-related paralysis or death, *n* = 3. **g**, **h** NSG-c-Kit mice were grafted with 5 × 10^6^ CACO2 human colon cancer cells subcutaneously on one flank. One month later, when tumor sizes reached 200 to 300 mm^3^, mice received i.v. injection of 1 × 10^7^ pfu of either WT VSV^IN^-IL-12 or Pro-VSV^IN^-IL-12 (Day 0). Mice were sacrificed 8 days post treatment, followed by collection of tumor tissue and indicated organs which were examined for the level of scIL-12 mRNA by RT-qPCR (**h**), *n* = 5. **i**, **j** C57BL/6J mice were injected i.v. with 200 μl of PBS, 1 × 10^7^ pfu of WT VSV^IN^-IL-12, or 1 × 10^7^ pfu of Pro-VSV^IN^-IL-12 twice, three days apart. Mouse serum was collected one day following the last injection and analyzed for IL-12p70 (**I**) and IFN-γ (**J**) by multiplex ELISA. **k**–**r** 5 × 10^5^ MC38 cells were grafted subcutaneously to one flank of C57BL/6J mice. On days 7, 10, 13, and 16 post tumor graft, 5 × 10^6^ pfu of WT VSV^IN^-GFP, 1.875 × 10^7^ pfu of Pro-VSV^IN^-GFP, 5 × 10^6^ pfu of WT VSV^IN^-IL-12, 5 × 10^7^ pfu of Pro-VSV^IN^-IL-12, or PBS were injected i.v., as indicated by the pink arrows. Average tumor volumes (**k**), and individual tumor volumes over time for mice treated with PBS (**l**), WT VSV^IN^-GFP (**m**), Pro-VSV^IN^-GFP (**n**), WT VSV^IN^-IL-12 (**o**), and Pro-VSV^IN^-IL-12 (**p**). **q** Respective cure rates of each treatment determined by the number of mice achieving tumor eradication. **r** Kaplan–Meir survival curves of mice treated with recombinant viruses, *n* = 10–11. **s**, **t** MC38 tumor-bearing mice were treated with i.v. injected Pro-VSV^IN^-IL-12 to achieve tumor eradication. Around 1 to 1.5 months after the last treatment, mice were challenged with a subcutaneous injection of 5 × 10^5^ MC38 cells, and tumor growth was followed for 30 days (**s**), or sacrificed and 1 × 10^6^ splenocytes were harvested and co-cultured with 5 × 10^4^ MC38 tumor cells and supernatants were assayed 48 h later for IFN-γ and compared against splenocytes isolated from naïve control mice (**t**)

Both WT VSV^IN^ and Pro-VSV^IN^ were engineered to carry scIL-12 as a payload (WT VSV^IN^-IL-12 and Pro-VSV^IN^-IL-12, respectively) (Fig. 5a, b). TEM of both showed the classic VSV bullet shape and size (~ 200 nm) (Fig. 5c, d; supplementary Fig. 6a–c). Viral G^IN^, N, and M protein expression, as well as the FLAG-tagged CR-2 domain connected to VSV-G^IN^ in the Pro-VSV^IN^-IL-12 virus were confirmed by immunoblot analysis (Fig. 5e). Additionally, to assess scIL-12 secretion, BHK-21 cells were transduced with either WT VSV^IN^-IL-12 or Pro-VSV^IN^-IL-12, and cell supernatant was tested in immunoblot analysis with an IL-12 p35-specific antibody (Fig. 5e). Further, the plaque diameter formed by WT VSV^IN^-IL-12 and Pro-VSV^IN^-IL-12 was decreased compared to their GFP expressing counterparts, indicating slower replication kinetics due to the IL-12 payload, which is larger in size than GFP (supplementary Fig. 6d).

To interrogate the safety profile of the IL-12 containing viruses, a dose escalation study in C57BL/6J hosts was performed. Pro-VSV^IN^-IL-12 is safe at a dose 10-fold higher than WT VSV^IN^-IL-12, since no treatment related paralysis, or death was observed following systemic injection of Pro-VSV^IN^-IL-12 at titers up to 2 × 10^8^ pfu per injection, compared to 2 × 10^7^ pfu for WT VSV^IN^-IL-12 (Fig. 5f). A biodistribution study over eight days using CACO2 tumor bearing NSG-c-KIT mice showed the preference of Pro-VSV^IN^-IL-12 to accumulate in tumor, compared to that of WT VSV^IN^-IL-12, which also accumulated in liver (Fig. 5g, h). When injected i.v. to immune competent C57BL/6J mice, WT VSV^IN^-IL-12 led to significant elevation in IL-12 and IFN-γ protein levels in the serum (Fig. 5i, j). This effect was largely abolished in the case of Pro-VSV^IN^-IL-12 (Fig. 5i, j). It is known that IL-12 is toxic when systemically exposed, and the reduction in IL-12 and IFN-γ exposure in the serum by Pro-VSV^IN^-IL-12 agrees with its enhanced safety profile.

Next, to explore the potential therapeutic benefit of Pro-VSV^IN^-IL-12 as a cancer treatment, we established a syngeneic, ectopic model of colon adenocarcinoma by grafting MC38 cells subcutaneously to the flanks of C57BL/6J mice (Fig. 5k–r). The tumors were palpable 7 days following the tumor graft, at which time the mice were treated with four doses every three days by i.v. injection of PBS or one-quarter of the determined MTD of the transgenic viruses. While little or no tumor control was observed for mice treated with PBS (Fig. 5l, supplementary Fig. 6e), WT VSV^IN^-GFP (Fig. 5m, supplementary Fig. 6f), as well as Pro-VSV^IN^-GFP (Fig. 5n, supplementary Fig. 6g), the addition of the potent immune-stimulating scIL-12 in both WT VSV^IN^-IL-12 (Fig. 5o, supplementary Fig. 6h) and Pro-VSV^IN^-IL-12 (Fig. 5p, supplementary Fig. 6i) resulted in delayed tumor growth. However, unlike WT VSV^IN^-IL-12, Pro-VSV^IN^-IL-12 proved to be the superior treatment, eradicating tumors in nine of ten mice, and significantly increasing the survival of mice compared to all other treatment groups (Fig. 5p–r). Importantly, no treatment-related deaths or signs of toxicity were observed in the Pro-VSV^IN^-IL-12 treated cohort. Additionally, to test if treatment with Pro-VSV^IN^-IL-12 conferred long-term immune memory, Pro-VSV^IN^-IL-12 treated mice were rechallenged with MC38 tumor cells 40–60 days after the initial tumor graft. None of the 9 mice grew tumors following MC38 subcutaneous rechallenge (Fig. 5s). Immune memory was further confirmed by isolating splenocytes from mice with tumors eradicated following treatment with Pro-VSV^IN^-IL-12, co-culturing with MC38 tumor cells, and analyzing for the expression of IFN-γ (Fig. 5t).

To explore the scope of anti-tumor efficacy of Pro-VSV^IN^-IL-12, we treated tumor-bearing mice with four doses of PBS or Pro-VSV^IN^-IL-12 by intraperitoneal (i.p.), instead of i.v., injection every three days (supplementary Fig. 6j–o). Treatment with Pro-VSV^IN^-IL-12 resulted in a significant decrease in the growth rate of tumors compared to PBS treated controls, and eradicated tumors in seven of ten mice (supplementary Fig. 6j–l). Likewise, Pro-VSV^IN^-IL-12 significantly extended the lives of mice compared to PBS treated controls (supplementary Fig. 6m). As observed with i.v. administration of Pro-VSV^IN^-IL-12, i.p. administration likewise conferred immune memory (supplementary Fig. 6p).

**Fig. 6:**
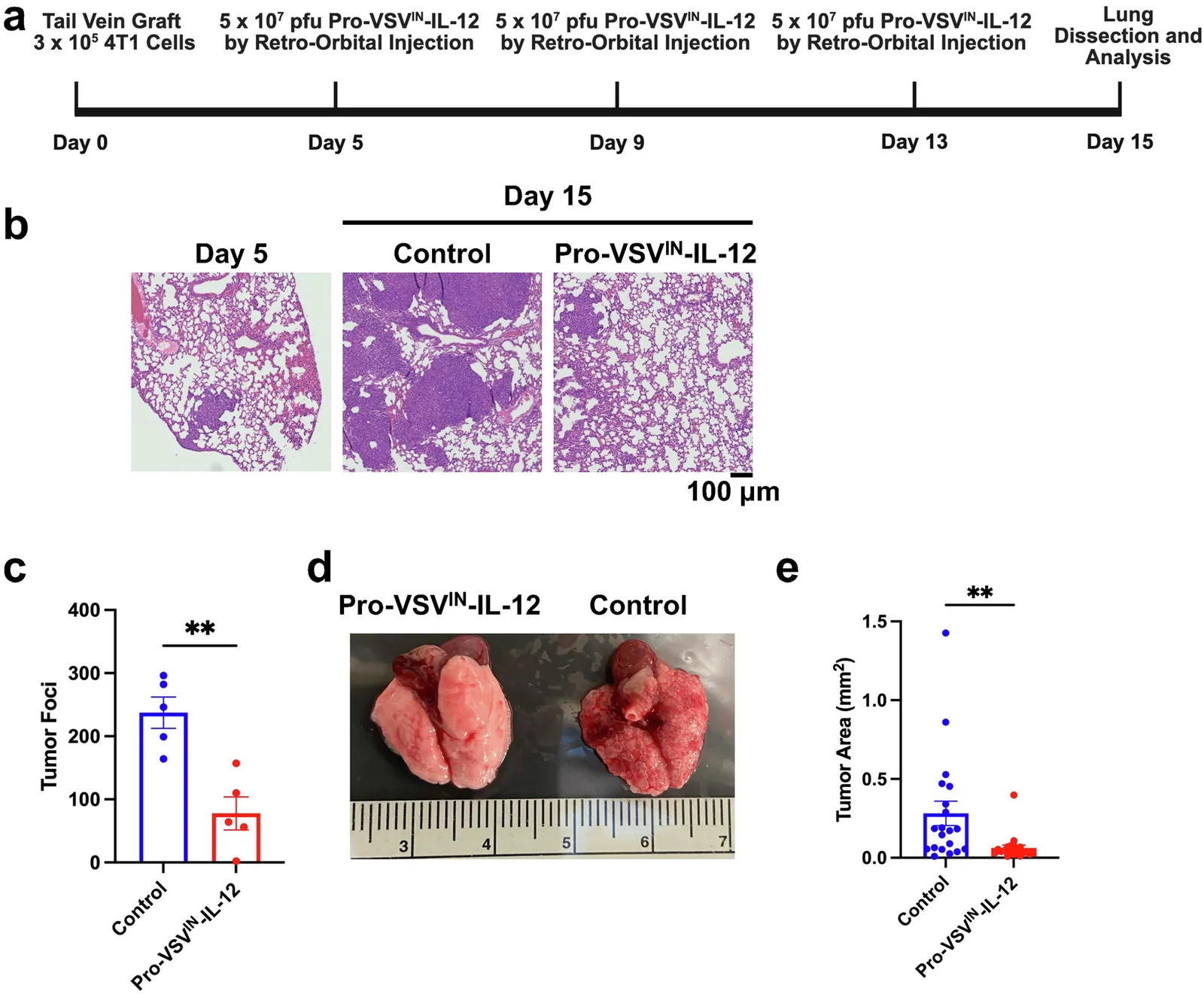
Systemic Pro-VSV^IN^-IL-12 is effective against metastatic breast cancer. **a**–**e** 3 × 10^5^ 4T1 cells were grafted to lungs of BALB/c mice by tail vein injection (Day 0). 5 days after tumor cell injection, mouse lungs were fixed and embedded in paraffin, followed by sectioning, H/E staining, and analyzed to confirm the presence of tumor nodules prior to treatment with virus (**b**, left panel). On days 5, 9, and 13 post tumor cell injection, 5 × 10^7^ pfu of Pro-VSV^IN^-IL-12 or PBS (control) were injected i.v. to the treatment groups (**a**). On day 15, mice were sacrificed and their lungs were analyzed for the presence of tumor nodules by H/E staining on sectioned lungs (**b**, right two panels), visual examination and quantification of metastatic nodules on the surface of the lungs (**c**, **d**), and quantification on the sizes of metastatic nodules on H/E-stained lung sections (**e**)

Finally, to test the scope of Pro-VSV^IN^-IL-12 efficacy, 4T1 breast cancer cells were grafted into BALB/c mice via tail vein injection to allow for the formation of lung metastasis (Fig. 6a). 5 days later, at which time the lung metastatic nodules were already established (Fig. 6b, left panel), mice were treated with Pro-VSV^IN^-IL-12 or PBS. Results showed that systemic Pro-VSV^IN^-IL-12 injection significantly reduced metastatic lung nodules with no signs of toxicity (Fig. 6c, d). Further, H/E staining showed that the remaining metastatic nodules in Pro-VSV^IN^-IL-12 treated group were significantly smaller in sizes compared to the control group (Fig. 6b, middle and right panels, e).

### Systemic Pro-VSV^IN^-IL-12 activates tumor-infiltrating T cells

**Fig. 7:**
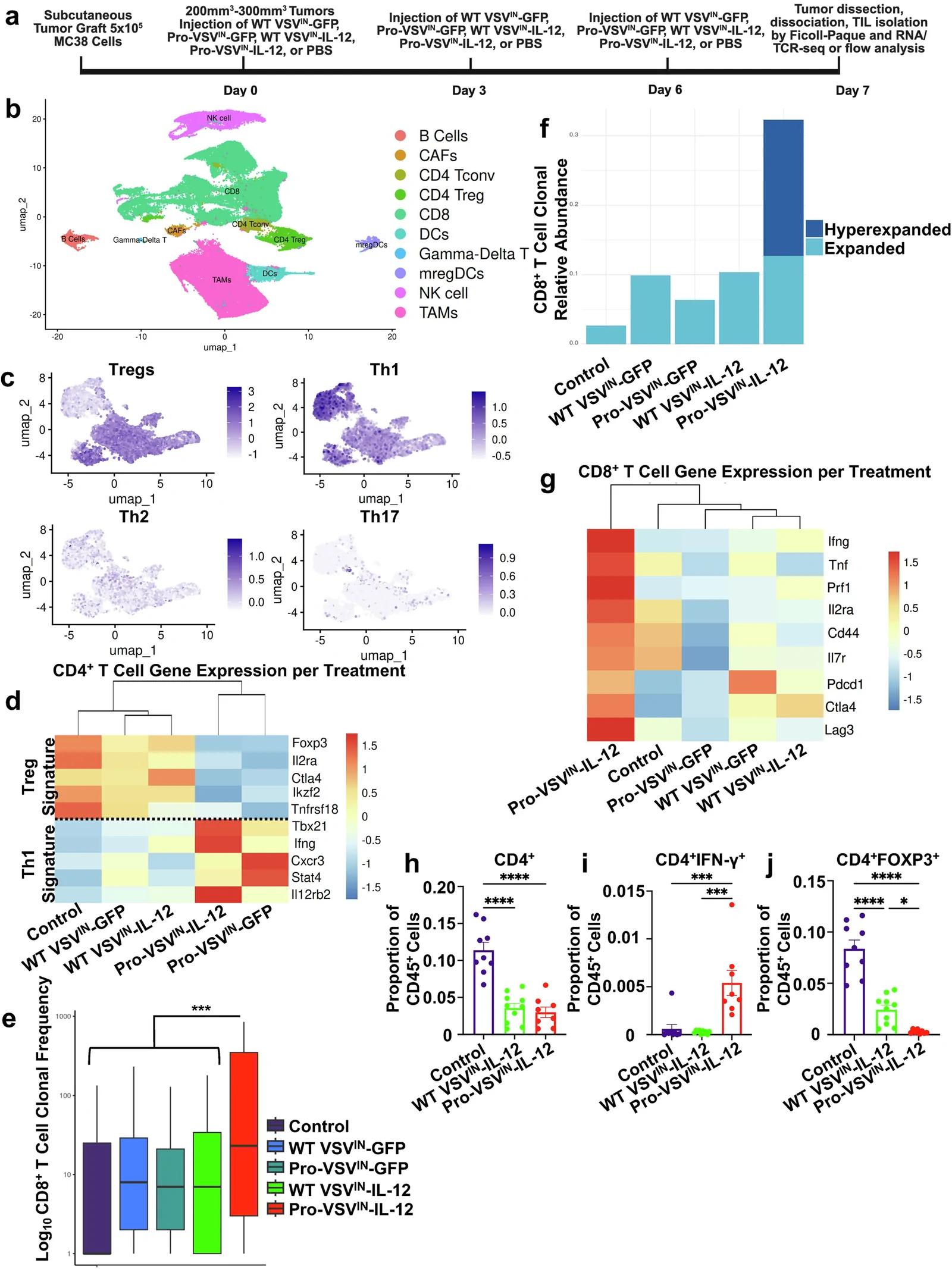
Systemic treatment with Pro-VSV^IN^-IL-12 depletes Tregs and bolsters Th1s in the TME. **a** Schematic for the analysis of tumor infiltrating lymphocytes (TILs) by flow cytometry or single-cell RNA or TCR sequencing. 5 × 10^5^ MC38 cells were grafted subcutaneously to the flanks of C57BL/6J mice. When tumors reached a size of 200–300 mm^3^, mice received i.v. injection of 5 × 10^6^ pfu of WT VSV^IN^-GFP, 1.875 × 10^7^ pfu of Pro-VSV^IN^-GFP, 5 × 10^6^ pfu of WT VSV^IN^-IL-12, and 5 × 10^7^ pfu of Pro-VSV^IN^-IL-12, or PBS on days 0. Two additional treatments were administered on days 3 and 6, followed by tumor dissociation, and analysis of TILs by flow cytometry or sequencing on day 7. **b** Merged UMAP of cell types captured by sequencing. Cell type assignments were determined based on expression of markers highlighted in supplementary Fig. 7. **c** CD4^+^ T cell subsets (CD4^+^ T conventional cells plus CD4^+^ Tregs) were reclustered and analyzed for expression of CD4 T cell subset markers. FeaturePlots showing module scores for each Treg subset. Module scores were created using the R package Seurat (see “Materials and methods” for details). **d** Heatmap showing the module scores for Treg and Th1 CD4^+^ T cell subsets across each experimental condition. **e** Boxplot shows the distribution of clonal frequency (number of matching clones for each unique TCR) for CD8^+^ T cells from 10x Genomics TCR (VDJ) sequencing. In each plot, the box represents the interquartile range, the horizontal line inside the box indicates the median. **f** Proportion of expanded (0.01 < X ≤ 0.05) and hyperexpanded (0.05 < X ≤ 1) CD8^+^ T cell clones out of total CD8^+^ T cells. TCR data analyzed using scRepetoire and Immunarch. **g** Heatmap of CD8^+^ T cells highlighting markers of CD8^+^ T cell activation and effector function. Proportion of CD45^+^ cells was analyzed for CD4^+^ (**h**), CD4^+^IFN-γ^+^ (**i**), and CD4^+^FOXP3^+^ (**j**) T cell populations by flow cytometry

To elucidate the mechanism of action of Pro-VSV^IN^-IL-12, we investigated the tumor-infiltrating lymphocyte (TIL) populations impacted by systemic treatment with Pro-VSV^IN^-IL-12. To do so, we implanted MC38 tumor cells into the flanks of mice, and treated mice with three doses of PBS, WT VSV^IN^-GFP, Pro-VSV^IN^-GFP, WT VSV^IN^-IL-12, or Pro-VSV^IN^-IL-12 (Fig. 7a). One day after the last injection, TILs were isolated by Ficoll-Paque density centrifugation and analyzed by single-cell RNA and TCR sequencing, as well as flow cytometry (Fig. 7b–j, supplementary Fig. 7). Across all treatment conditions, we observed a remodeling of the CD4^+^ T cell compartment, with the greatest change observed in Treg and Th1 cell populations (Fig. 7c, h–j). Specifically, treatment with Pro-VSV^IN^-IL-12 resulted in the greatest increase in the expression of Th1-associated genes, and a downregulation of genes associated with Tregs (Fig. 7d). The shift towards Th1 signature genes was a result of the increased proportion of Th1 cells, and decreased proportion of Tregs, in the tumor (supplementary Fig. 8a, b). Th1 cells within MC38 tumors of the Pro-VSV^IN^-IL-12 treated mice further show enhanced expression of IFN-γ and T-bet (supplementary Fig. 8c, d). Consistent with prior literature on the effects of IL-12 on Treg stability and suppressive function,^73,74^ Tregs in Pro-VSV^IN^-IL-12 treated tumors showed increased levels of T-bet (supplementary Fig. 8e, f). These Treg cells also show markedly reduced level of CD103 (encoded by *Itgae*) expression, suggesting a deficit in Treg migration into the tumor that could also contribute to the observed changes in Treg number (supplementary Fig. 8g). The reduction in Tregs and increase in Th1 cells following Pro-VSV^IN^-IL-12 treatment was further validated by flow cytometry (Fig. 7h–j).

In the CD8^+^ T cell compartment, treatment with Pro-VSV^IN^-IL-12 resulted in the greatest clonal expansion compared to all other treatment groups (Fig. 7e, supplementary Fig. 9a). Interestingly, only TIL from Pro-VSV^IN^-IL-12 treated mice contained CD8^+^ T cell clones which were hyperexpanded (Fig. 7f, supplementary Fig. 9a). These hyperexpanded CD8^+^ T cells express enriched markers for T cell effector function (supplementary Fig. 9b). When all CD8^+^ T cells (expanded or not) from each treatment group were compared, differential gene expression analysis revealed that administration of Pro-VSV^IN^-IL-12 resulted in the greatest increase in activation markers in CD8^+^ T cells (Fig. 7g). Moreover, monocytes had greater expression of antigen presentation and activation genes, and in the NK cell subsets higher levels of activation and effector molecule expression (supplementary Fig. 10a, b).

To find the cell population that mediates Pro-VSV^IN^-IL-12 efficacy, CD4, CD8, and NK cells were depleted with antibodies in MC38 tumor-bearing mice treated with Pro-VSV^IN^-IL-12. CD8^+^ T cell depletion diminished efficacy, but NK cell depletion did not alter the outcome of the therapy (supplementary Fig. 10c, d). Interestingly, we also did not observe a change in Pro-VSV^IN^-IL-12’s effectiveness following ablation of CD4^+^ T cells (supplementary Fig. 10c, d). This may be due to the depletion of both CD4^+^ Th1 cells and Treg cells by the anti-CD4 antibody. It is known that these two T cell populations play contradicting roles in cancer immunotherapy. Taken together, we show that systemic injection of Pro-VSV^IN^-IL-12 activates Th1 and CD8^+^ T cells, and reduces Treg cells in the tumor environment.

### Combination therapy of single dose Pro-VSV^IN^-IL-12 with checkpoint blockade improves anti-tumor potency

**Fig. 8:**
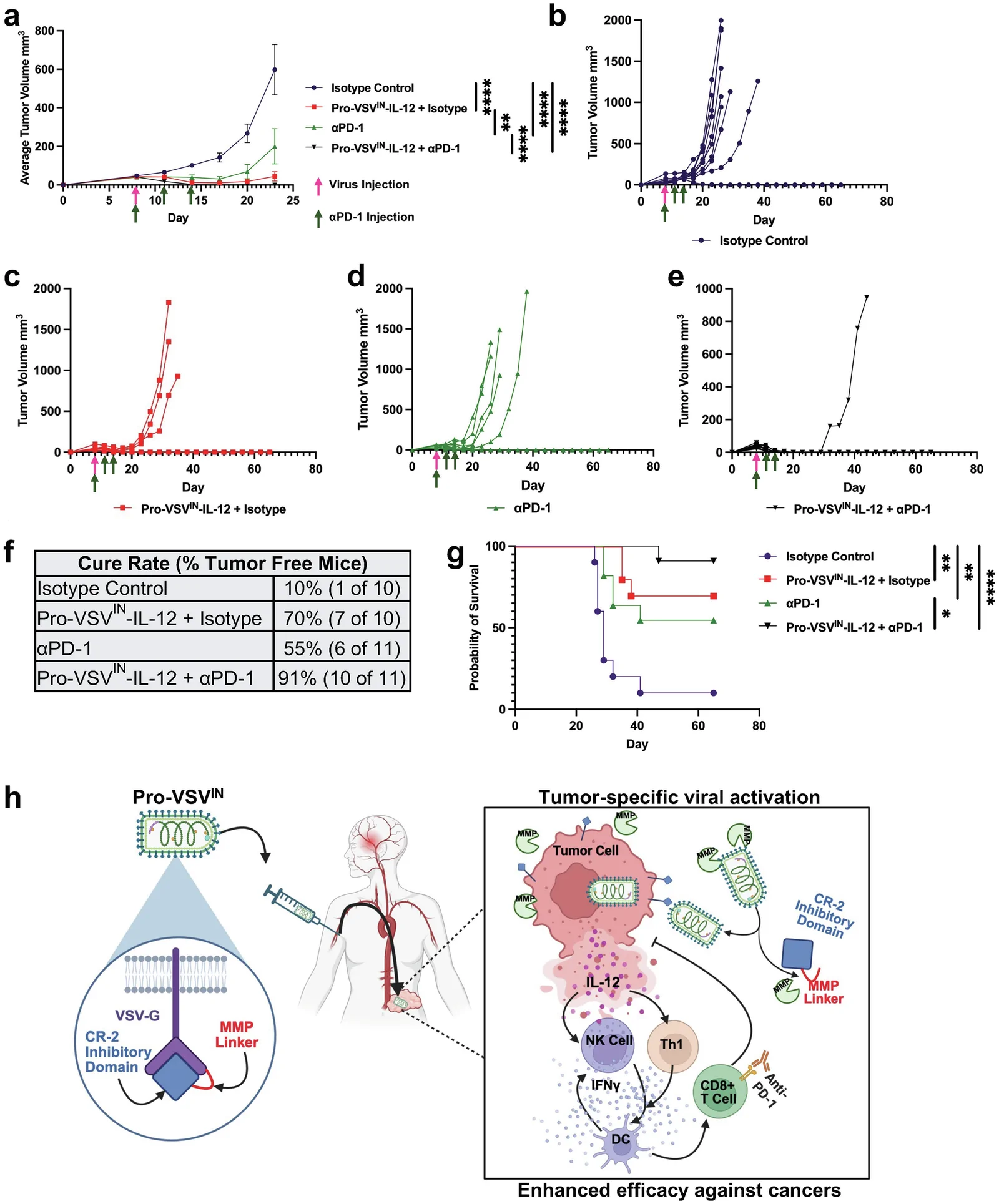
Single dose treatment of Pro-VSV^IN^-IL-12 combined with anti-PD-1 eradicates tumors. 5 × 10^5^ MC38 cells were subcutaneously grafted to one flank of C57BL/6J mice. On day 8 post tumor graft, when tumors were an average of 40–50 mm^3^, mice were treated with a single dose i.v. injection of Pro-VSV^IN^-IL-12 (5 × 10^7^ pfu) combined with intraperitoneal (i.p.) injection of αPD-1 (100 μg/200 μl) or an isotype control (100 μg/200 μl), αPD-1 alone, or isotype control alone. Mice received two additional treatments, on day 11 and 14, of αPD-1 or isotype control by i.p. injection. **a**–**e** Average tumor volumes (**a**), and individual tumor volumes over time for mice treated with (**b**) isotype control, (**c**) Pro-VSV^IN^-IL-12 + isotype, (**d**), αPD-1, and (**e**) Pro-VSV^IN^-IL-12 + αPD-1. Pink arrows mark the injection of virus, and green arrows indicate administration of αPD-1. **f** Respective cure rates of each treatment determined by the number of mice achieving tumor eradication. **g** Kaplan–Meir survival curves of mice for each treatment group, *n* = 10–11. **h** Design of Pro-VSV and its cancer-specific targeting mechanism. The Pro-VSV-G^IN^-MMP guided vesicular stomatitis virus (Pro-VSV^IN^), which is dormant in its native form, is selectively activated in the tumor microenvironment (TME) by matrix metalloproteinases (MMPs) expressed by tumor cells. Through this mechanism, Pro-VSV^IN^ is preserved in systemic circulation and selectively infects and amplifies within tumor tissues. Localized delivery of immune activating agents, such as IL-12, by Pro-VSV^IN^ results in activation of Th1-armed immunity and CD8^+^ T cells in the TME, permitting effective anti-cancer therapy with limited toxicity compared to systemic delivery of recombinant IL-12 and other immune modulating agents

Immune checkpoint blockade has become a standard of care for several cancer types. To test if treatment with the combination of Pro-VSV^IN^-IL-12 and αPD-1 would further the therapeutic benefit of checkpoint blockade, we grafted MC38 cells to the flanks of mice, and when tumors reached an average volume of 40–50 mm^3^, treated mice with a single dose of Pro-VSV^IN^-IL-12 with αPD-1 (Fig. 8a–g, supplementary Fig. 11). The combination of Pro-VSV^IN^-IL-12 with checkpoint blockade outperformed isotype treated controls (Fig. 8b), a single dose of Pro-VSV^IN^-IL-12 with isotype control treated mice (Fig. 8c), as well as αPD-1 as a monotherapy (Fig. 8d), eradicating tumors in all but one treated mouse (Fig. 8e). Additionally, combination of Pro-VSV^IN^-IL-12 and αPD-1 led to significantly improved long-term survival of treated mice, with no tumor regrowth following the cure of these established colon tumors (Fig. 8f, g). Overall, our data suggest that the cap-linker strategy specifically targets Pro-VSV^IN^ to the tumor environment (Fig. 8h). The tumor-restricted delivery of therapeutic payloads, such as IL-12, contributes to the greatly enhanced safety and efficacy for the Pro-VSV^IN^ platform. Systemic administration of Pro-VSV^IN^-IL-12 potently activates anti-cancer immunity in the tumor, and improves the outcome of immune checkpoint blockade.

## Discussion

To date, two virotherapies have been FDA approved in the United States. One is T-Vec, an oncolytic virus which was approved in 2015 for unresectable melanoma and the second is Adstiladrin, a non-oncolytic virus, approved in 2022 for non-muscle invasive bladder cancer. Currently, no oncolytic viruses have been approved for systemic injection, but multiple therapies are under clinical evaluation including a modified VSV that carries IFN-β.^75^ Development of systemically injected oncolytic viral agents requires careful evaluation of their payloads to avoid intolerable immune-related adverse events. For instance, IL-12 was incorporated into several viruses including HSV,^76^ adenovirus,^77^ and vaccinia virus (clinical trial NCT04301011). Clinical trials with these viruses used intratumoral injection as a delivery method. In our study, we show that the cap-linker modification on the VSV-G^IN^ protein significantly enhanced the safety margin of IL-12 armed VSV. Compared to WT VSV^IN^ that carries IL-12, Pro-VSV^IN^-IL-12 has a 10-times higher MTD (Fig. 5f). Further, when injected at the same dose, Pro-VSV^IN^ showed undetectable distribution to normal organs such as the liver and the brain, and enhanced replication in grafted tumors in mice (Figs. 4k, 5h). These promising results demonstrate the enhanced tumor-specificity of the Pro-VSV platform, allowing i.v. administration for the treatment of surgically unreachable tumors.

We systematically tested the feasibility of different CR domains from human LDL-R as the inhibitory domain (the cap), which allowed for the robust rescue of viral activity following cleavage of the linker to VSV-G^IN^ (Fig. 2). Both CR-2 and CR-3 domains physically interact with VSV-G^IN^,^19^ and indeed, when the CR-2 domain was mutated on four amino acids that were reported to mediate its binding to VSV-G,^IN^ the signal-to-noise ratio of the mutant cap-VSV-G^IN^ protein in guiding viral infection was diminished (Fig. 1e, f). To maximize these findings, we propose that our cap-linker strategy may be applied to other viruses and their cognate receptor domains, whereby the binding activity between the cap and viral spike protein serves as a key for effective inhibition of viral entry until the linker between them is cleaved.

We chose IL-12 as a payload to be tested in this study due to its potency in inducing anti-tumor immunity. Recombinant IL-12 has been tested in human cancers, but failed a phase II clinical trial due to the loss of two patients,^55,78^ indicating the need for more targeted delivery methods of IL-12. Pro-VSV^IN^-IL-12 addresses this problem due to its enhanced tumor-specificity compared to WT VSV^IN^. Selective replication of Pro-VSV^IN^-IL-12 provides sustained local secretion of the potent Th1 inducing cytokine. In addition to IL-12, Pro-VSV may encode other cytokines, chemokines, antibodies, and tumoricidal proteins. For instance, bispecific antibodies against tumor-associated antigens and CD3 serve as T cell engagers (TCEs) to “pull” human T cells into contact with tumor cells, resulting in potent tumor killing. One major issue with systemic delivery of recombinant TCEs is the expression of the selected tumor antigens in normal organs, leading to “on target, off tumor” activation of T cells and severe toxicity in some cases.^79^ Specific delivery of T cell engagers by Pro-VSV may allow enhanced therapeutic tolerance and efficacy for human cancers, which remains to be interrogated.

Our Pro-VSV design relies on the enzymatic activity of MMPs in the tumor environment to cleave the linker and release the inhibition to VSV-G^IN^. The MMP family contains more than 20 members and are widely upregulated and overexpressed in human cancers.^47,80^ Therefore, the potential application of the Pro-VSV platform, and Pro-VSV^IN^-IL-12 in particular, should not be limited to colorectal and breast cancers as tested in this study, but to all solid tumors with elevated MMP activity. On the other hand, it is possible that heterogeneity in MMP expression level/activation exists among cancer patients. Therefore, it may be feasible to start future clinical evaluation of Pro-VSV^IN^-IL-12 in patients whose tumor biopsies show upregulated MMP expression. In addition to MMPs, human tumors also overexpress other proteases, and often exhibit reduced pH levels compared to normal tissues.^81,82^ Exploiting these characteristics of human tumors may aid further modification of the Pro-VSV by varying the linker region of the Pro-VSV^IN^-G protein to respond to acidic environments or other proteases such as matriptase and hepsin.^83^

In addition to limited tumor specificity, systemic injection of oncolytic viral therapies also faces induction of host immunity against viral proteins, and varying efficiency of viruses reaching the tumor site. It is unclear if the masking of VSV-G^IN^ by the human LDL-R CR-2 domain alleviates immunogenicity of Pro-VSV compared to its WT counterpart. In mice, repeated Pro-VSV^IN^-IL-12 was done in multiple cancer models, and was effective in controlling tumor growth (Figs. 5 and 6). The treatment scheme spans around two weeks of time, and within this period we did not observe obvious loss of therapeutic activity against tumors. The impact of host immunity, such as antibodies against VSV-G^IN^ protein, remains to be tested in a human clinical setting. Another approach to enhance viral entry into the tumor site is the delivery of viruses along other vehicles, such as T cells.^84^ Previously, we tested the co-delivery of VSV-IFN-β with CAR-T cells and showed marked increase in tumor viral titer compared to i.v. injected VSV alone.^84^ A similar approach may be employed to further enhance the tumor accumulation of Pro-VSV to the tumor sites.

In this study, we show that Pro-VSV^IN^-IL-12 has much higher MTD compared to WT VSV^IN^-IL-12. Thanks to this enhanced safety profile, we were able to treat mice with a much higher dose of Pro-VSV^IN^-IL-12 in our therapeutic efficacy study, which resulted in almost complete eradication of established tumors. However, this treatment scheme makes it difficult to distinguish improved intrinsic anti-tumor potency from dose-dependent effects of Pro-VSV^IN^-IL-12. A future study comparing equal dose of Pro-VSV^IN^-IL-12 with WT VSV^IN^-IL-12 will help clarify this point.

To best imitate a clinical setting, we combined Pro-VSV^IN^-IL-12 with immune checkpoint inhibitors to test for therapeutic efficacy. Combination therapy eradicated the majority of grafted MC38 tumors, surpassing both monotherapies using αPD-1 or Pro-VSV^IN^-IL-12 (Fig. 8). The single injection of Pro-VSV^IN^-IL-12 in this treatment scheme, when translated to human clinical practice, would also circumvent the potential caveat of virus-induced immunity. Mechanistically, we show that Pro-VSV^IN^-IL-12 potently activates Th1 cells and CD8^+^ T cells in the tumor environment. Several T cell activation/exhaustion markers were upregulated in T cells following Pro-VSV^IN^-IL-12 i.v. treatment, including PD-1. Significantly increased proportion of Th1 cells in the tumor also contributes to the secretion of IFN-γ, which is known to induce PD-L1 expression in tumor cells.^85^ These data support our observed synergy between Pro-VSV^IN^-IL-12 and αPD-1. Other combination schemes are also under investigation, and our understanding on how Pro-VSV^IN^-IL-12 remodels tumor immunity may inform their chance of success. For example, we also observed that Pro-VSV^IN^-IL-12 potently reduced the population of Tregs in the tumor. Inhibition and depletion of Tregs was thought to be one of the major mechanisms of action by anti-CTLA4 blocking antibody. On the other hand, since Pro-VSV^IN^-IL-12 fosters an immunologically “hot” environment in the tumor, combining this i.v. oncolytic viral therapy with other immune checkpoints, T cell co-stimulation agonists, and other immunotherapeutic regimens that require T cell infiltration as a prerequisite, may further enhance the potential of developing effective therapies against advanced human cancers that fail checkpoint blockade or chemotherapy.

## Materials and methods

### Cells

MC38 murine colon adenocarcinoma cells were obtained from Millipore Sigma (Cat # SCC172), HEK-293T and BHK-21 cells were obtained from the American Type Culture Collection (ATCC, CRL-11268 and CCL-10, respectively), 4T1 cells were obtained from Dr. Weizhou Zhang at the University of Florida, and maintained in DMEM (Corning Cat #10013CV) supplemented with 5% or 10% FBS (Corning, Cat #35011CV) and 1% penicillin-streptomycin (Gibco, Cat #15140122) at 37 °C with 5% CO_2_. C2-11 MMP-KO HEK-293T cells were derived from HEK-293T cells by CRISPR/Cas9. Primary human brain microvascular endothelial cells (pBEC) were obtained from Dr. Patrik Murphy at UConn Health, and maintained in Vascular Cell Basal Medium (ATCC, Cat #PCS-1000-30) supplemented with 2% FBS, 5 ng/ml human VEGF, 5 ng/ml human EGF, 5 ng/ml human FGF, 15 ng/ml human IGF, 10 mM L-glutamine, 0.75 units/ml Heparin, 1 μg/ml hydrocortisone, and 50 μg/ml ascorbic acid.

### Matrix metalloproteinase edited HEK-293T cells using CRISPR/Cas9 and analysis of gene knockout efficiency on a DNA level

HEK-293T cells (1.5 × 10^5^) were electroporated with a 9:1 sgRNA to Cas9 ratio using Lonza’s SF Cell Line 4D-Nucleofector^®^ X Kit (Cat# V4XC-2032) program CM-130, following the manufacturer’s protocol. All sgRNA sequences are listed in Table 1. All guide RNAs were purchased as ready Gene-Knockout Kits V2 from Synthego.

**Table 1:** sgRNA sequences for CRISPR/Cas9 MMP gene knockout

Gene target	sgRNA sequences (5’–3’)	Forward primer	Reverse primer	Sequencing primer
MMP2	1. CUGGGGCGAAGGCAUGAGCC 2. GACGAUGAGCUAUGGACCUU 3. UGUGUUUCAGAGCAUGGCGA	GCTCCTGTTGACAGTCCTGG	GACATGGAAGGGGGAGGTTG	CTCCTGTTGACAGTCCTGGATAAC
MMP11	1. GGCGUGCCCGACCCAUCUGA 2. CCUCUCGGCAUGGAGGUGGU 3. CUGCCACGCAGGAAGCCCCC	GTGCCCTTTCCCTCTCCATT	ACGAAGATCTACCCTGGCCT	CTTTCCCTCTCCATTTGCCCAG
MMP15	1. UCAUGGCGCCGUUCUACCAG 2. AUGCCACGGAGAUCGUCCUC 3. AGGAAACAACCUCUUCCUGG	GGAGGAGAGAGTTCCCCTGT	AACCTGTGTCCCTTCAAGGC	GATCACTACACTCAATTTCAGTCTGTTGTC
MMP16	1. UCAGGGAGCAGCCCAGGCAG 2. GGAGCAGUAUUUCAAUGUGG 3. UCACAUUCAGCACUGGAAGA	CGGGGATAACGGATTCTGGG	TGTCTCCTCCCCTTGCC	CATCCGTCACCTAAAACACCCTTAAAC
MMP17	1. ACUCAUGUACUACGCCCUCA 2. CGGCGCUGCCCGCCACCUCG 3. CGAAGGGGUAGCCGUCGUUA	AGGAGGGGCGTTCAGGGAA	CTGCTCCTCAGCCTCACAG	AGGAGGGGCGTTCAGGGAAC

Following electroporation, pooled HEK-293T cells were sorted for single colony selection using Becton-Dickinson FacsAria II cell sorter at the UConn Health Flow Cytometry Core Facility. Following clonal expansion, genomic DNA was extracted using DNeasy^®^ Blood & Tissue Kit (Qiagen, Cat# 69504), and target regions were amplified by PCR using KOD Hot Start Polymerase (Sigma-Aldrich, Cat# 71086-3), verified by agarose gel electrophoresis prior to purification of PCR products using the QIAquick Gel Extraction Kit (Qiagen, Cat# 28706). PCR amplicons were Sanger sequenced and paired ab1 files of control versus edited samples were analyzed using Synthego’s ICE tool (https://ice.editco.bio/#/). PCR and sequencing primers (purchased from IDT) used are listed in Table 1.

### Virus construction and production

The second-generation lentiviral system was utilized to investigate VSV-G^IN^, VSV-G^NJ^ and Maraba-G spike proteins and modifications made to the spike proteins.^86^ VSV-G^IN^ (Addgene plasmid #138479) was cloned into the pCDNA3-TOPO vector (a gift from Dr. Jianbin Ruan, Department of Immunology, UConn Health) for purposes of lentiviral production, in conjunction with psPAX2 (Addgene #12260), and pLenti-eGFP (Addgene plasmid #141395). The pcDNA3-TOPO vector was used as the parental vector for cloning.

For the generation of lentivirus, adherent HEK-293T or C2-11 MMP-KO HEK-293T cells were used and seeded to be ~70% confluent at the time of transfection, typically 3 × 10^5^ cells per well of a 6-well plate, on plates coated with Poly-D-Lysine (Gibco, Cat # A3890401). Manufacturer’s protocol was used for transfection. Briefly, at the time of transfection, Lipofectamine^TM^ 3000 (Invitrogen, Cat # L30000015) was diluted in 125 μl of Opti-MEM^TM^ I Reduced Serum Medium (Gibco, Cat # 31985070), and combined with 2.5 μg of total DNA of psPAX2, pLenti-GFP and spike protein containing pCDNA3-TOPO plasmids, diluted in 125 μl Opti-MEM^TM^, and combined with P3000 reagent, then mixed and incubated at room temperature for 15 min before being added to the cells in a drop-wise manner. Cells containing the transfection complex were incubated for 24 h at 37 °C in 5% CO_2_ prior to medium change to Opti-MEM^TM^. Viral supernatant was harvested 48 h post transfection and clarified and filtered prior to use.

WT HEK-293T cells or C2-11 MMP-KO HEK-293T cells were transduced with GFP-expressing lentivirus. Cells were harvested 72 h post transduction and stained for viability using Live/Dead fixable exclusion dye and then analyzed for the presence of GFP. Flow cytometry analyses were performed on BD FACSymphony A5 SE flow cytometer at the UConn Health Flow Cytometry Core Facility. Data were analyzed with FlowJo software (FlowJo, LLC).

All VSV constructs were generated from the recombinant pVSV eGFP ΔG plasmid (Addgene plasmid # 31842).^87^ VSV-G^IN^ or Pro-VSV-G^IN^ were cloned into the fifth (ΔG) position using MluI and NotI restriction enzyme cut sites. A single-chain (sc) Interleukin (IL)-12 (scIL-12),^88^ harboring mutated MscI restriction enzyme cut sites (Twist Biosciences) was cloned into the first (GFP) position using XhoI and MscI restriction enzyme cut sites. WT or Pro-VSV^IN^ expressing GFP or scIL-12 were rescued from the VSV cDNA plasmid using preestablished reverse genetics systems in BHK-21 cells, as previously described.^89–92^ In brief, BHK-21 cells were infected with MVA-T7 (a gift from Dr. Richard Vile) at an MOI of 1, incubated at 37 °C and 5% CO_2_ for 1 h, then transfected with pVSV genomic plasmid (10 μg), pBluescript (pBS)-encoding VSV-N (3 μg), pBS-encoding VSV-P (5 μg), and pBS-encoding VSV-L (1 μg) proteins (pBS plasmids were a gift from Dr. Richard Vile) using Fugene6 (Promega, Cat # E2691). After 48–72 h, supernatant was collected then clarified through a 0.2 μm filter. At least two rounds of plaque purification were performed.

Amplification of rescued VSV was achieved by a single round of infection of BHK-21 cells. Viral supernatant was collected after 24–96 h, clarified using a 0.2 μm filter and centrifuged at 20,000 × *g* for 3.5 h. The pellet was resuspended in an appropriate volume of phosphate-buffered saline (PBS) (Corning, Cat # 21-040-CM). Viral titers were determined by standard plaque assay of serially diluted samples using BHK-21 cells. For titering of Pro-VSV^IN^ viruses, an additional step of digesting Pro-VSV^IN^ with active MMP-7 protease (MilliporeSigma, Cat # 444270) was performed for 1 h at 4 °C followed by the standard plaque assay procedure.

### Animal models

C57BL/6J, NOD.Cg-Kit^W-41J^ Tyr^+^ Prkdc^scid^ Il2rg^tm1Wjl^/ThomJ (JAX stock # 026622),^93–95^ and BALB/c mice were obtained from the Jackson Laboratory. All mice were maintained in specific-pathogen-free conditions with filter-topped cages on autoclaved food and water at UConn Health, and all experiments were approved by the IACUC of UConn Health and performed in accordance with the university and NIH guidelines and regulations. All experiments used co-housed, gender-matched littermates.

### Determination of maximum tolerated dose

The MTD for this study is defined as the highest dose of virus administered not resulting in paralysis or death. Male and female C57BL/6J mice were given a single intravenous (retro-orbital) dose of WT VSV^IN^-GFP, Pro-VSV^IN^-GFP, WT VSV^IN^-IL-12, or Pro-VSV^IN^-IL-12 ranging from 2 × 10^6^ pfu to 3 × 10^8^ pfu, and monitored up to two months post injection for any clinical signs. Dose levels of each virus were increased incrementally until any clinical signs were observed in injected mice, at which point dose escalation was ceased and MTD set.

### Biodistribution studies

NSG-c-Kit mice were grafted with 5 × 10^6^ CACO2 human colon cancer cells subcutaneously on one flank. When tumors reached a volume of 200 to 300 mm^3^, mice were treated with a single i.v. (retro-orbital) dose of 1 × 10^7^ plaque forming units (pfu) of each transgenic virus suspended in 200 μl of PBS. Mice were sacrificed at indicated timepoints of 0, 1, 2, 4, and 8 days post injection. Major organs, tissues, and tumors were harvested, weighed, and analyzed for the presence of viral RNA by RT-qPCR.

### Tumor immunotherapy models

MC38, or 4T1 cells were cultured in DMEM with 5 or 10% FBS, respectively, penicillin, and streptomycin. Cultured MC38 cells were digested with trypsin, washed, and resuspended at a concentration of 5 × 10^5^ cells per 50 μl in 1x PBS. To establish subcutaneous tumors, cells were injected into the right flanks of 2-to-3-month-old C57BL/6J (for MC38) mice. For metastatic models, 4T1 at a concentration of 3 × 10^5^ cells per 200 μl in PBS were grafted by tail vein injection to the lungs of BALB/c mice. Equal numbers of male and female mice were used for the tests, and mice were assigned randomly into different treatment groups. Viral injections (5 × 10^6^–5 × 10^7^ pfu suspended in 200 μl of PBS) were performed intravenously (by retro-orbital injection) or intraperitoneally at indicated timepoints after tumor grafting. Tumor length and width were measured every three days with a caliper. Tumor volume was calculated as V = (Length × Width^2^)/2. Mice were euthanized when tumors reached a volume of 2 × 10^3^ mm^3^.

For checkpoint blockade experiments, mice were given 100 μg αPD-1 antibody (Bio X Cell, Cat # BP0146) or isotype control (Bio X Cell, Cat # BP0089) which were injected intraperitoneally at indicated timepoints.

For in vivo immune memory experiments, mice treated with Pro-VSV^IN^-IL-12 whose tumors were eradicated, were rechallenged by a subcutaneous graft of 5 × 10^5^ MC38 cells into their right flank. In some cases, a second round rechallenge was performed by injecting the same number of MC38 cells to the opposite flank of mice 3 months after the first rechallenge. Naïve C57BL/6J mice were used as controls.

For in vitro immune memory experiments, splenocytes were isolated from mice treated with Pro-VSV^IN^-IL-12 whose tumors were eradicated, or from naïve mice, and resuspended at a concentration of 1 × 10^6^ cells/mL in DMEM containing 5% FBS and 1% penicillin-streptomycin. These splenocytes were co-cultured with 5 × 10^4^ MC38 tumor cells for 48 h, and supernatants were analyzed for IFN-γ by an enzyme-linked immunosorbent assay (ELISA) per manufacturer’s instructions (Mabtech, Cat # 3321-1H-6 and # 3652-F10).

### Antibody depletion

Depleting antibodies against CD4 (Bio X Cell, Cat # BE0003), CD8α (Bio X Cell, Cat # ΒΕ0061), and NK1.1 (Bio X Cell # BE0036) were injected intraperitoneally at 200 μg at indicated timepoints. The control antibodies (Bio X Cell # BE0083 and BP0089) were administered to the other groups. Flow analysis of splenocytes was used to confirm subset-specific depletions.

### Tumor infiltrating immune cell isolation and flow cytometry analysis

Tumor infiltrating immune cells were isolated as previously described.^96,97^ In brief, tumors were isolated from the flanks of mice, mechanically dissociated using scissors and crushed through a 70 μm cell strainer in a single well of a 6-well plate to generate a single cell suspension. The cell suspension was then passed through a second 70 μm cell strainer into a 50 ml conical tube and diluted up to a total volume of 30 ml with RPMI-1640 media. Next, 10 ml of Ficoll-Paque^TM^ PLUS (GE Healthcare, Cat # 17-1440-03) was slowly added to the bottom of the 50 ml conical tube, and then centrifuged at 1025 × *g* for 20 min at 20 °C. Following centrifugation, the upper 20 ml layer of media was discarded, and the remaining 10 ml of media and layer of mononuclear cells were isolated and transferred to a clean 50 ml conical tube. Complete RPMI-1640 medium was then used to wash the mononuclear cells twice at 650 × *g* for 10 min. Upon completion of the washes, cells were stimulated using a Cell Stimulation Cocktail (eBioscience, Cat # 00-4970-03) for 3 h, stained with Live/Dead fixable exclusion dye (Tonbo Bioscience, Cat# 13-0868), followed by fluorochrome-conjugated antibodies in PBS with 2% fetal bovine serum and 1 mM EDTA for 1 h at 4 °C for anti-CD45.2 (eBioscience, Cat # 17-0454-82), anti-CD45 (Biolegend, Cat # 103138), anti-CD3 (Tonbo Bioscience, Cat # 75-0032), anti-CD4 (Biolegend, Cat # 100453), and anti-CD8α antibody (Biolegend Cat # 100751 and Tonbo Bioscience, Cat # 20-0081-U025). Upon completion of surface marker staining, cells were washed twice followed by fixation with a Foxp3/transcription factor staining buffer set (eBioscience, Cat# 00-5523-00) and intracellular staining for anti-IFN-γ (Biolegend, Cat #505806), and anti-Foxp3 antibody (Invitrogen, Cat # 12-5773-82). Flow cytometry analyses were performed on BD FACSymphony A5 SE flow cytometer at the UConn Health Flow Cytometry Core Facility. Data were analyzed with FlowJo software (FlowJo, LLC).

### RT-qPCR analysis

Total RNA was extracted with RNeasy Plus Kit to eliminate genomic DNA contamination (Qiagen, Cat# 74134) and reverse transcribed using an iScript Kit (Biorad, Cat # 1708891). RT-qPCR was performed using SsoAdvanced Universal SYBR Green Supermix (Biorad, Cat # 1725275) on the CFX96 Real-Time PCR detection system (Biorad). Expression data were normalized to mouse or human GAPDH mRNA levels. The data were calculated as 2^(Ct(GAPDH–gene of interest))^ to compare experimental groups to controls, and are presented in arbitrary units. Primer sequences are listed in Table 2 (purchased from IDT). Whenever possible, primers were intron-spanning, such that amplification was only feasible on complementary DNA.

**Table 2:** Primer sequences for RT-qPCR

mRNA	Reverse primer 5’–3’	Forward primer 5’–3’
Mouse GAPDH	GAAGGGGTCGTTGATGGCAA	TGGTGAAGGTCGGTGTGAAC
Human GAPDH	CAGAGTTAAAAGCAGCCCTGGT	GAAGGTGAAGGTCGGAGTCAAC
GFP	GAAGATGGTGCGCTCCTG	AGTGCTTCAGCCGCTACC
scIL-12	CTGGAATGACCCTAGATCCGC	CCGAAGTCCAATGCAAAGGC

### Serum cytokine analysis

C57BL/6J mice were treated with 1 × 10^7^ pfu of WT VSV^IN^-IL-12 or Pro-VSV^IN^-IL-12 every three days for a total of two treatments. One day following the last treatment, blood was collected from mice, left at room temperature to clot for 30 min, and centrifuged at 1000 × *g* for 15 min followed by serum collection. Serum cytokine analysis was run targeting IFN-γ and IL-12p70 according to manufacturer’s specification (BioLegend, LEGENDplex Cat # 740824).

### Histology of lung tissue

Lungs were harvested and fixed in 10% formalin in PBS for 48 h at 4 °C, washed with PBS, then dehydrated in 70% ethanol in water followed by paraffin-embedding, and sectioning. H&E-stained sections were prepared for analysis of metastatic nodules at the UConn Health Histology Core Facility.

### Transmission electron microscopy (TEM) imaging of the viral particles

Concentrated virus particles were fixed using 1% glutaraldehyde solution for 10 min on ice. 10 μl of the fixed viral particles were placed on glow discharged 400 mesh carbon-coated nickel grid (Electron Microscopy Sciences, #CF400NI50) for 1 min. Grids were then rinsed with PBS (3 times, 5 min each) and further with DI water (3 times, 5 min each). Negative stain with freshly prepared 1% uranyl acetate in DI water was applied to the grids for 45 s. The grids were let to dry and examined under Hitachi H7650 Transmission Electron Microscopy at the UConn Health Central Electron Microscopy Facility.

### Western blotting

Antibodies used for Western blotting include: Anti-VSV-G^IN^ antibody (Sigma-Aldrich, Cat # V4888), Anti-VSV-G^NJ^ (a gift from CaroGen Corporation), Anti-FLAG M2 antibody (Millipore Sigma, Cat # F1804), Anti-VSV antibody (Imanis Life Sciences, Cat # REA005), IL-12p35 antibody/IL-12A (NSJ Bioreagents, Cat # RQ4058), Anti-rabbit IgG, HRP-linked Antibody (Cell Signaling Technology, Cat # 7074), Anti-mouse IgG, HRP-linked Antibody (Cell Signaling Technology, Cat # 7076).

### Single-cell RNA-Seq data processing and analysis

Tumors from 5 mice were dissected and tumor infiltrating lymphocytes (TILs) isolated and pooled. TILs were stained for CD45, CD3, CD4, CD8 and viability by propidium iodide (Invitrogen, Cat # P3566). After FACs, cells were washed and resuspended in PBS containing 0.04% BSA and immediately processed for sequencing as follows. Cells were counted and viability was assessed using the LUNA-FX7 (Logos Biosystems) with Acridine Orange/Propodium Iodide Stain. Up to 29,000 cells were then loaded for capture onto one lane of a 10x Genomics Chromium GEM-X Single Cell 5’ Chip v3. Capture, barcoding and llumina sequencing library preparation were performed according to manufacturer protocols using the 10x Genomics Chromium X platform 5’ version 3 GEM-X chemistry (protocol CG000733, revA). From each sample, cDNA was used for construction of both gene expression and TCR (VDJ) libraries. cDNA and libraries were assessed for quality via Tapestation 4200 (Agilent) and Qubit Fluorometer (ThermoFisher) and quantified by KAPA qPCR. Libraries were sequenced on an Illumina NovaSeq X+ (10B 100 cycle) flow cell with a 28-10-10-90 (R1-I1-I2-R2) asymmetric read configuration, targeting 20,000 barcoded cells with an average sequencing depth of 50,000 reads per cell and 15,000 reads per cell for the gene expression and TCR libraries, respectively. Sequencing was performed by the Jackson Laboratory Single-Cell Genomics Lab and University of Connecticut Center for Genome Innovation.

Illumina basecall (.bcl) files were converted to FASTQ using bcl2fastq v2.20.0.422 (Illumina) and FASTQ files were aligned to the GRCm39 reference assembly with vM33 annotations from GENCODE (10x Genomics GRCm39 reference version 2024-A) using 10X Genomics Cell Ranger ‘multi‘ pipeline (version 9.0.1) with default parameters. Single-cell RNA sequencing cell-counts matrices were analyzed using Seurat v5.3.0 with R version 4.5.1. A merged Seurat object containing each of the 5 conditions was generated, and QC was performed; retaining cells with ≥500 genes and ≤10% reads mapping to mitochondrial genes. Doublets were filtered using DoubletFinder version 2.0.6. A total of 88,297 single cells were retained after QC. Gene expression was normalized, scaled, and dimensionality reduction was performed. UMAP and heatmap plots were generated using Seurat, pheatmap v1.0.13 and ggplot2 v3.5.2. Single-cell TCR data was integrated with 5’ GEX data and analyzed using Seurat and scRepertoire v2.4.0. Clonal expansion was determined based on CDR3 nucleotide sequences using scRepertoire. Differential gene expression was determined using Seurat’s FindMarkers function (adjusted *p*-value < 0.05, log2 Fold Change ≥0.25, min.pct ≥0.1).

### Statistical analysis

Statistical analysis was performed with GraphPad Prism version 10.3.1 (GraphPad Software, San Diego, USA), unless stated otherwise. Multi-group comparison was analyzed by one-way ANOVA. Multi-group and multi-variable comparison was analyzed by two-way ANOVA. Pairwise comparison was analyzed by Student’s t-test. In all statistical tests, *p* < 0.05 was considered significant. In all experiments, *p < 0.05; **p < 0.01; ***p < 0.001; and ****p < 0.0001. Error bars denote SEM unless otherwise stated in the figure legends. Survival data from the animal studies were analyzed by log-rank test. Unless stated otherwise, *n* is the number of biological replicates (individual mice, organs, or tissue samples, etc.) with all experiments being repeated at least two times. Representative results are shown.

## Supplementary information

Supplementary Materials

Change of author form

## Data Availability

The data generated in this study are available upon request from the corresponding author. Raw and processed data from single-cell RNA sequencing were deposited at the Gene Expression Omnibus (GEO) with accession number GSE310378.
