# Supplementary material for: Matrix metalloproteinase-guided targeting of vesicular stomatitis virus to tumors following systemic administration
Source: Signal Transduct Target Ther. 2026 Oct 20;11:455. doi: 10.1038/s41392-026-02888-x (PMC13680497; doi:10.1038/s41392-026-02888-x)
Supplement: Supplementary file 1 [file 41392_2026_2888_MOESM2_ESM.pdf]

Change of authorship request form - Journals (pre-acceptance)

Important information. Please read.

- This form should be used by authors to request any change in authorship (adding/deleting authors) including changes in corresponding authors. This form should not be used for name changes. Please fully complete all sections. Use black ink and block capitals and provide each author's full name with the given name first followed by the family name.

- By signing this declaration, all authors guarantee that the order of the authors are in accordance with their scientific contribution, if applicable as different conventions apply per discipline, and that only authors have been added who made a meaningful contribution to the work.

- Please note, in author collaborations where there is formal agreement for representing the collaboration, it is sufficient for the representative or legal guarantor (usually the corresponding author) to complete and sign the Authorship Change Form on behalf of all authors, **next to the added/removed author(s)**. **(Complete Section 3, followed by Section 6.)**
  In author collaborations where there is no formal agreement for representing the collaboration and **there are more than 10 authors**, one may sign for all, provided the signer appends correspondence that attests that each of the authors have agreed to the change **and the added/removed authors sign the form**. **(Complete Section 3, followed by Section 6.)**

- Please note, we cannot investigate or mediate any authorship disputes. If you are unable to obtain agreement from all authors (including those who you wish to be removed) you must refer the matter to your institution(s) for investigation. Please inform us if you need to do this.

- If you are not able to return a fully completed form within **30 days** of the date that it was sent to the author requesting the change, we may have to withdraw your manuscript. We cannot publish manuscripts where authorship has not been agreed by all authors (including those who have been removed).

- Incomplete forms will be rejected.

- Please return/upload this form, fully completed, to the Journals Editorial Office. The Journal and/or Publisher will consider the information you have provided to decide whether to approve the proposed change in authorship. We may decide to contact your institution for more information or undertake a further investigation, if appropriate, before making a final decision.

SPRINGER NATURE

Change of authorship request form - Journals (pre-acceptance)

Section 1: Please provide the current title of manuscript

Manuscript ID no.: SIGTRANS-21404R

Title: MMP-Guided Targeting of Vesicular Stomatitis Virus to Tumors Following Systemic Administration

Section 2: Please provide the previous authorship, in the order shown on the manuscript before the changes were introduced. Please indicate the corresponding author by adding (CA) behind the name.

	First name(s)	Family name	ORCID or SCOPUS id, if available
1st author	Eileen	Theune	
2nd author	William	Theune	
3rd author	Elham	Ahmadi	
4th author	Conner	Chiari	
5th author	Neha	Singh	
6th author	Rory	Gayer	
7th author	Jason	Tonne	
8th author	Muriel	Metko	
9th author	Isabella	Wang	
10th author	Dylan	Dai	

Please use an additional sheet if there are more than 10 authors.

Jianbin	Ruan
Robert	Clark
Penghua	Wang
Evan	Jellison
Anthony	Vella
Richard	Vile
Kepeng	Wang -- CA

SPRINGER NATURE Springer Nature is one of the world's leading global research, educational and professional publishers, created in May 2015 through the combination of Nature Publishing Group, Palgrave Macmillan, Macmillan Education and Springer Science+Business Media.

Change of authorship request form - Journals (pre-acceptance)

Section 3: Please provide a justification for change. Please use this section to explain your reasons for changing the authorship of your manuscript, e.g. what necessitated the change in authorship? Please refer to the (journal) policy pages for more information about authorship. Please explain why omitted authors were not originally included and/or why authors were removed on the submitted manuscript.

We added Olamide T. Olaoba and Dr. Guangfu Li as contributing authors during the first revision of the manuscript. These two authors assisted in a portion of the experiments that were conducted during the revision. There is no change in the first or last author for the paper.

Section 4: Proposed new authorship. Please provide your new authorship list in the order you would like it to appear on the manuscript. Please indicate the corresponding author by adding (CA) behind the name. If the Corresponding Author has changed, please indicate the reason under section 3.

	First name(s)	Family name (this name will appear in full on the final publication and will be searchable in various abstract and indexing databases)	Affiliated institute	E-mail address
1st author	Eileen	Theune		
2nd author	William	Theune		
3rd author	Elham	Ahmadi		
4th author	Conner	Chiari		
5th author	Neha	Singh		
6th author	Rory	Gayer	Jianbin	Ruan
7th author	Jason	Tonne	Robert	Clark
8th author	Muriel	Metko	Guangfu	Li
9th author	Olamide	Olaoba	Penghua	Wang
10th author	Isabella	Wang	Evan	Jellison

Please use an additional sheet if there are more than 10 authors.

Dai

Dylan

Anthony Vella

Richard Vile

Kepeng Wang -- CA

SPRINGER NATURE Springer Nature is one of the world's leading global research, educational and professional publishers, created in May 2015 through the combination of Nature Publishing Group, Palgrave Macmillan, Macmillan Education and Springer Science+Business Media.

Change of authorship request form - Journals (pre-acceptance)

Section 5: Author contribution, Acknowledgement and Disclosures. Please use this section to provide a new disclosure statement and, if appropriate, acknowledge any contributors who have been removed as authors and ensure you state what contribution any new authors made (if applicable per the journal or book (series) policy). Please ensure these are updated in your manuscript - after approval of the change(s) - as our production department will not transfer the information in this form to your manuscript.

New acknowledgements: This work was supported by a National Institutes of Health training grant (T90DE033006) to E.V.T, National Institutes of Health/National Cancer Institute (R01CA282775) and National Heart Lung and Blood Institute (R01HL172239) to A.T.V., National Institutes of Health/National Cancer Institute (R01CA269384, R01AI170535 and CA210964-06) to R.V., and (R01CA262430, R01CA303989) to K.W. The content is solely the responsibility of the authors and does not necessarily represent the official views of the National Institutes of Health. The funders had no role in study design, data collection, and interpretation, or the decision to submit the work for publication.
We thank Dr. Mina Mina and Dr. Ali Tamayol for their generous support through the T90 training grant to E.V.T. We also thank Dr. Weizhou Zhang (University of Florida) for 4T1 cells, Dr. Patrick Murphy (UConn Health) for primary brain microvascular endothelial cells, and CaroGen Corporation for VSV-G New Jersey antibody. We thank Dr. Li Zhu at the UConn Health Flow Cytometry Core Facility, and Dr. Zhifang Hao at the UConn Health Histology Core Facility for technical assistance, as well as Li Chen and Dr. David Rowe at the UConn Health Center for Regenerative Medicine and Skeletal Development for imaging assistance, as well as Dr. Bernard L. Cook III at the UConn School of Medicine for scientific editing. We also thank the contribution of the Single Cell Biology service, the Genome Technologies service, and Cyberinfrastructure high performance computing resources at The Jackson Laboratory for expert assistance with the work described herein whose shared services are supported in part by the JAX Cancer Center (P30 CA034196). We also thank Kira V. Theune for her devoted presence and support throughout the final stages of this project. Schematic graphics were generated with Biorender.com.

New Disclosures (financial and non-financial interests, funding): E.V.T. and K.W. are inventors of a provisional patent application (US application No. 63/800843) related to this work. K.W. is the founder of Symunology, Inc. The authors declare no additional financial interest.

New Author Contributions statement (if applicable per the journal policy):E.V.T and K.W. conceived the project. E.V.T., W.C.T, E.A., C.C.C., N.S., R.G., J.T., M.M., O.T.O., I.C.W., and D.D. performed the experiments and analyzed the data. K.W., R.V., J.R., R.C., G.L., P.W., E.R.J., and A.T.V. provided conceptual advice and analyzed the data. E.V.T. and W.C.T. drew the figures, E.V.T. and K.W. wrote the manuscript, with all authors contributing to the writing, editing, and providing key advice. All authors have read and approved the article.

State 'Not applicable' if there are no new authors.

SPRINGER NATURE

Change of authorship request form - Journals (pre-acceptance)

Section 6: Declaration of agreement. ***All*** **authors, unchanged, new and removed** ***must*** **sign this declaration.**
(NB: Please print the form, (docu)-sign and return/upload a scanned copy. Please note that signatures that have been inserted as an image file are acceptable as long as it is handwritten. Typed names in the signature box are unacceptable.) *** Please delete as appropriate. Delete all of the bold if you were on the original authorship list and are remaining as an author.**

	First name	Family name		Signature	Date
1st author	Eileen	Theune	I agree to the proposed new authorship shown in section 4 /and the addition/removal*of my name to the authorship list /and the proposed change in corresponding author		5.22.26
2nd author	William	Theune	I agree to the proposed new authorship shown in section 4 /and the addition/removal*of my name to the authorship list /and the proposed change in corresponding author		5.22.26
3rd author	Ethan	Ahmadi	I agree to the proposed new authorship shown in section 4 /and the addition/removal*of my name to the authorship list /and the proposed change in corresponding author		5/22/2026
4th authors	Conner	Chiari	I agree to the proposed new authorship shown in section 4 /and the addition/removal*of my name to the authorship list /and the proposed change in corresponding author		5/22/2026
5th author	Neha	Singh	I agree to the proposed new authorship shown in section 4 /and the addition/removal*of my name to the authorship list /and the proposed change in corresponding author	Neha	5/26/26
6th author	Rory	Geyer	I agree to the proposed new authorship shown in section 4 /and the addition/removal*of my name to the authorship list /and the proposed change in corresponding author		5/26/26
7th author	Jason	Tonne	I agree to the proposed new authorship shown in section 4 /and the addition/removal*of my name to the authorship list /and the proposed change in corresponding author		5/26/26

SPRINGER NATURE Springer Nature is one of the world's leading global research, educational and professional publishers, created in May 2015 through the combination of Nature Publishing Group, Palgrave Macmillan, Macmillan Education and Springer Science+Business Media.

SPRINGER NATURE

Change of authorship request form - Journals (pre-acceptance)

Section 6: Declaration of agreement. *All* authors, unchanged, new and removed *must* sign this declaration.
(NB: Please print the form, (docu)-sign and return/upload a scanned copy. Please note that signatures that have been inserted as an image file are acceptable as long as it is handwritten. Typed names in the signature box are unacceptable.) *** Please delete as appropriate. Delete all of the bold if you were on the original authorship list and are remaining as an author.**

	First name	Family name		Signature	Date
8 author	Muriel	Metko	I agree to the proposed new authorship shown in section 4 ~~/and the addition/removal*of my name to the authorship list /and the proposed change in corresponding author~~		
9 author	Olamide	Olaoba	I agree to the proposed new authorship shown in section 4 **/and the addition~~/removal*of my~~ name to the authorship list** ~~/and the proposed change in corresponding author~~		
3 author	Isabella	Wang	I agree to the proposed new authorship shown in section 4 ~~/and the addition/removal*of my name to the authorship list /and the proposed change in corresponding author~~	[signature]	5/25/26
1 authors	Dylan	Dai	I agree to the proposed new authorship shown in section 4 ~~/and the addition/removal*of my name to the authorship list /and the proposed change in corresponding author~~	[signature]	5/25/26
2 author	Jianbin	Ruan	I agree to the proposed new authorship shown in section 4 ~~/and the addition/removal*of my name to the authorship list /and the proposed change in corresponding author~~	[signature]	5/22/26
3 author	Robert	Clark	I agree to the proposed new authorship shown in section 4 ~~/and the addition/removal*of my name to the authorship list /and the proposed change in corresponding author~~	Robert B Clark	5/22/26
4 author	Guangfu	Li	I agree to the proposed new authorship shown in section 4 **/and the addition~~/removal*~~of my name to the authorship list** ~~/and the proposed change in corresponding author~~	[signature]	5/20/26

SPRINGER NATURE Springer Nature is one of the world's leading global research, educational and professional publishers, created in May 2015 through the combination of Nature Publishing Group, Palgrave Macmillan, Macmillan Education and Springer Science+Business Media.

SPRINGER NATURE

Change of authorship request form - Journals (pre-acceptance)

Section 6: Declaration of agreement. *All* authors, unchanged, new and removed *must* sign this declaration.
(NB: Please print the form, (docu)-sign and return/upload a scanned copy. Please note that signatures that have been inserted as an image file are acceptable as long as it is handwritten. Typed names in the signature box are unacceptable.) *** Please delete as appropriate. Delete all of the bold if you were on the original authorship list and are remaining as an author.**

	First name	Family name		Signature	Date
8th author	Muriel	Metko	I agree to the proposed new authorship shown in section 4 ~~/and the addition/removal*of my name to the authorship list /and the proposed change in corresponding author~~	[signature] Muriel Metko	5/25/26
9th author	Olamide	Olaoba	I agree to the proposed new authorship shown in section 4 **/and the addition/**~~removal*of my~~ **name to the authorship list** ~~/and the proposed change in corresponding author~~	[signature]	5/25/26
10th author	Isabella	Wang	I agree to the proposed new authorship shown in section 4 ~~/and the addition/removal*of my name to the authorship list /and the proposed change in corresponding author~~		
11th authors	Dylan	Dai	I agree to the proposed new authorship shown in section 4 ~~/and the addition/removal*of my name to the authorship list /and the proposed change in corresponding author~~		
12th author	Jianbin	Ruan	I agree to the proposed new authorship shown in section 4 ~~/and the addition/removal*of my name to the authorship list /and the proposed change in corresponding author~~	[signature]	5/22/26
13th author	Robert	Clark	I agree to the proposed new authorship shown in section 4 ~~/and the addition/removal*of my name to the authorship list /and the proposed change in corresponding author~~	Robert B Clark	5/22/26
14th author	Guangfu	Li	I agree to the proposed new authorship shown in section 4 **/and the addition/**~~removal*~~**of my name to the authorship list** ~~/and the proposed change in corresponding author~~	[signature]	5/22/26

SPRINGER NATURE Springer Nature is one of the world's leading global research, educational and professional publishers, created in May 2015 through the combination of Nature Publishing Group, Palgrave Macmillan, Macmillan Education and Springer Science+Business Media.

SPRINGER NATURE

Change of authorship request form - Journals (pre-acceptance)

Section 6: Declaration of agreement. *All* authors, unchanged, new and removed *must* sign this declaration.
(NB: Please print the form, (docu)-sign and return/upload a scanned copy. Please note that signatures that have been inserted as an image file are acceptable as long as it is handwritten. Typed names in the signature box are unacceptable.) *** Please delete as appropriate. Delete all of the bold if you were on the original authorship list and are remaining as an author.**

	First name	Family name		Signature	Date
~~1st~~ 15 author	penghua	Wang	I agree to the proposed new authorship shown in section 4 ~~/and the addition/removal*of my name to the authorship list /and the proposed change in corresponding author~~	[signature]	5/22/2026
~~2nd~~ 16 author	Evan	Jellison	I agree to the proposed new authorship shown in section 4 ~~/and the addition/removal*of my name to the authorship list /and the proposed change in corresponding author~~		
~~3rd~~ 17 author	Anthony	Vella	I agree to the proposed new authorship shown in section 4 ~~/and the addition/removal*of my name to the authorship list /and the proposed change in corresponding author~~		
~~4th~~ 18 authors	Richard	Vile	I agree to the proposed new authorship shown in section 4 ~~/and the addition/removal*of my name to the authorship list /and the proposed change in corresponding author~~	[signature]	26/5/26
~~5th~~ 19 author	Kepeng	Wang	I agree to the proposed new authorship shown in section 4 ~~/and the addition/removal*of my name to the authorship list /and the proposed change in corresponding author~~	[signature]	5/22/2026
~~6th author~~			I agree to the proposed new authorship shown in section 4 **/and the addition/removal*of my name to the authorship list** /and the proposed change in corresponding author		
~~7th author~~			I agree to the proposed new authorship shown in section 4 **/and the addition/removal*of my name to the authorship list** /and the proposed change in corresponding author		

SPRINGER NATURE

Change of authorship request form - Journals (pre-acceptance)

Section 6: Declaration of agreement. *All* authors, unchanged, new and removed *must* sign this declaration.
(NB: Please print the form, (docu)-sign and return/upload a scanned copy. Please note that signatures that have been inserted as an image file are acceptable as long as it is handwritten. Typed names in the signature box are unacceptable.) *** Please delete as appropriate. Delete all of the bold if you were on the original authorship list and are remaining as an author.**

	First name	Family name		Signature	Date
~~1st~~ 15 author	Penghua	Wang	I agree to the proposed new authorship shown in section 4 ~~/and the addition/removal*of my name to the authorship list /and the proposed change in corresponding author~~	Wang Penghua	5/22/2026
~~2nd~~ 16 author	Evan	Jellison	I agree to the proposed new authorship shown in section 4 ~~/and the addition/removal*of my name to the authorship list /and the proposed change in corresponding author~~	Evan R Jellison<br>Digitally signed by Evan Jellison<br>DN: cn=Evan Jellison, o=UCONN Health, ou=Department of Immunology, email=jellison@uchc.edu, c=US<br>Date: 2026.05.25 08:14:14 -04'00'	
~~3rd~~ 17 author	Anthony	Vella	I agree to the proposed new authorship shown in section 4 ~~/and the addition/removal*of my name to the authorship list /and the proposed change in corresponding author~~	Anthony Vella<br>Digitally signed by Anthony Vella<br>Date: 2026.05.26 13:49:20 -04'00'	
~~4th~~ 18 authors	Richard	Vile	I agree to the proposed new authorship shown in section 4 ~~/and the addition/removal*of my name to the authorship list /and the proposed change in corresponding author~~		
~~5th~~ 19 author	Keperg	Wang	I agree to the proposed new authorship shown in section 4 ~~/and the addition/removal*of my name to the authorship list /and the proposed change in corresponding author~~	[signature]	5/22/2026
~~6th author~~			I agree to the proposed new authorship shown in section 4 **/and the addition/removal*of my name to the authorship list** /and the proposed change in corresponding author		
~~7th author~~			I agree to the proposed new authorship shown in section 4 **/and the addition/removal*of my name to the authorship list** /and the proposed change in corresponding author		

SPRINGER NATURE Springer Nature is one of the world's leading global research, educational and professional publishers, created in May 2015 through the combination of Nature Publishing Group, Palgrave Macmillan, Macmillan Education and Springer Science+Business Media.
