# Supplementary material for: Matrix metalloproteinase-guided targeting of vesicular stomatitis virus to tumors following systemic administration
Source: Signal Transduct Target Ther. 2026 Oct 20;11:455. doi: 10.1038/s41392-026-02888-x (PMC13680497; doi:10.1038/s41392-026-02888-x)
Supplement: Supplementary file 2 [file 41392_2026_2888_MOESM1_ESM.docx]

Supplementary Materials for

Matrix metalloproteinase-guided targeting of vesicular stomatitis virus to tumors following systemic administration

Eileen Victoria Theune, William C. Theune, Elham Ahmadi, Conner C. Chiari, Neha Singh, Rory Geyer, Jason Tonne, Muriel Metko, Olamide T. Olaoba, Isabella China Wang, Dylan Dai, Jianbin Ruan, Robert Clark, Guangfu Li, Penghua Wang, Evan R. Jellison, Anthony T. Vella, Richard Vile, and Kepeng Wang.

Correspondence to: kewang@uchc.edu

**This PDF file includes:**

Figures. S1 to S11

**Supplementary Fig. 1: Inhibition and rescue of viral activity of VSV-G^IN^ spike protein is greatest with the LDL-R CR-2 domain, related to Figure 2. a,** Fluorescent cell imaging of HEK-293T cells transduced with GFP-expressing lentivirus pseudotyped with WT VSV-G^IN^ or Pro-VSV-G^IN^ harboring CR-1 to CR-7 domains as inhibitory domains. Viruses were added to cells in their native form or following 3C protease digest for 3 h on ice prior to infection. **b,** Analysis of viral genome copies by GFP mRNA level by RT-qPCR, normalized to GAPDH. **c,** Western blot detection of VSV-G^IN^ and FLAG, before and after 3C protease digest to confirm linker cleavage and inhibitory domain release.

**Supplementary Fig. 2:** **Inhibition and rescue of viral activity of VSV-G^NJ^ spike protein is greatest with the LDL-R CR-2 domain, related to Figure 2. a,** Fluorescent cell imaging of HEK-293T cells transduced with GFP-expressing lentivirus pseudotyped with WT VSV-G^NJ^ or Pro-VSV-G^NJ^ harboring CR-1 to CR-7 as inhibitory domains. Viruses were added to cells in their native form or following 3C protease digest for 3 h on ice prior to infection. **b,** Analysis of viral genome copies by GFP mRNA level by RT-qPCR, normalized to GAPDH. **c,** Western blot detection of VSV-G^NJ^ and FLAG, before and after 3C protease digest to confirm linker cleavage and inhibitory domain release.

**Supplementary Fig. 3:** **Inhibition and rescue of viral activity with Maraba-G spike protein is greatest with the LDL-R CR-4 domain, related to Figure 2.** **a,** Fluorescent cell imaging of HEK-293T cells transduced with GFP-expressing lentivirus pseudotyped with WT Maraba-G or Pro-Maraba-G harboring CR-1 to CR-7 as inhibitory domains. Viruses were added to cells in their native form or following 3C protease digest for 3 h on ice prior to infection. **b,** Analysis of viral genome copies by GFP mRNA level by RT-qPCR, normalized to GAPDH. **c,** Western blot detection of FLAG, before and after 3C protease digest to confirm linker cleavage and inhibitory domain release.

**Supplementary Fig. 4: CRISPR knockout of MMPs in HEK-293T cells allows for efficient production of Pro-VSV-G^IN^-MMP pseudotyped lentivirus, related to Figure 3**. **a,** Analysis of gene expression levels of MMPs in HEK-293T cells by RT-qPCR, normalized to human GAPDH, n = 3. **b,** Schematic for the generation of MMP-KO HEK-293T single clone “C2-11” cells by electroporation of sgRNAs and CRISPR/Cas9 proteins targeting MMPs 2, 11, 15, 16, and 17. These genes were targeted sequentially, confirmed to be knocked out by sanger sequencing of genomic DNA, with single cell clone selection to identify the best candidate. **c,** Synthego ICE analysis of C2-11 MMP-KO HEK-293T cells generated by CRISPR/Cas9 gene knockout of MMPs 2, 11, 15, 16, and 17. **d,** Illustration of the comparison of C2-11 MMP-KO HEK-293T cells against WT HEK-293T cells (“WT cells”) for their efficiency of viral production following transfection. **e,** Analysis of viral genome copies by GFP mRNA level by RT-qPCR for WT VSV-G^IN^, Pro-VSV-G^IN^-3C (harboring a 3C cleavable linker), and Pro-VSV-G^IN^-MMP (harboring a MMP cleavable linker) pseudotyped lentiviruses produced in WT cells and C2-11 cells, n = 9. **f,** Schematic for the comparison of WT cells against C2-11 cells for their efficiency in viral transduction by indicated viruses, including the transduction of Pro-VSV-G^IN^-MMP pseudotyped lentivirus undigested or digested with 0.3 U/ml MMP-7 protease for one hour at 4 °C. **g,** Quantification of virally infected cells 72 h post transduction by flow cytometry, n = 3.

**Supplementary Fig. 5:** **Pro-VSV^IN^ Spike Protein Modification De-Targets Liver, related to Figure 4. a,** NSG-c-Kit mice were grafted with 5 x 10^6^ CACO2 human colon cancer cells subcutaneously on one flank. One month later, when tumor sizes reached 200 to 300 mm^3^, mice received i.v. injection of 1 x 10^7^ pfu of Pro-VSV^IN^-GFP (Day 0). Mice were sacrificed at indicated time points post treatment, followed by collection of tumor tissue and indicated organs which were examined for the level of GFP mRNA by RT-qPCR, n = 2. **b,** CACO2 tumor bearing NSG-c-Kit mice received an i.v. injection of PBS, or 1 x 10^7^ pfu of either WT VSV^IN^-GFP or Pro-VSV^IN^-GFP (Day 0). Mice were sacrificed 8 days post treatment. Mouse livers were cryosectioned and stained by H/E.

**Supplementary Fig. 6: Pro-VSV^IN^-IL-12 is an effective anti-tumor therapy when administered by intraperitoneal injection, related to Figure 5. a-b:** TEM at a 50,000x magnification with particle size analysis of **(a)** WT VSV^IN^-IL-12 and **(b)** Pro-VSV^IN^-IL-12. **c,** Quantification of TEM viral particle size analysis of WT VSV^IN^-IL-12 and Pro-VSV^IN^-IL-12, n = 7. **d,** Plaque diameter size determination of recombinant viruses by ImageJ generated measurements. **e-i:** Bodyweight tracking over time for MC38 tumor bearing mice treated with PBS **(e)**, WT VSV^IN^-GFP **(f)**, Pro-VSV^IN^-GFP **(g)**, WT VSV^IN^-IL-12 **(h)**, or Pro-VSV^IN^-IL-12 **(i)**. **j-o:** 5 x 10^5^ MC38 cells were subcutaneously grafted to one flank of C57BL/6J mice. On days 10, 13, 16, and 19 post tumor graft, mice were intraperitoneally injected with 5 x 10^7^ pfu of Pro-VSV^IN^-IL-12 or PBS. **j-l**: Average tumor volumes **(j)**, individual tumor volumes for mice treated with PBS **(k**), or Pro-VSV^IN^-IL-12 **(l)**. **m**, Kaplan-Meir survival curves of mice for each treatment group, n = 11-15. Bodyweight of control **(n)** and Pro-VSV^IN^-IL-12 **(o)** treated mice were tracked over time. **p,** MC38 tumor-bearing mice were treated with multiple doses of intraperitoneally injected Pro-VSV^IN^-IL-12 to achieve tumor eradication. 2 months after the last treatment, mice were rechallenged with a subcutaneous injection of 5 x 10^5^ MC38 cells, and signs of tumor growth was followed for 15 days. A second round of a subcutaneous injection of 5 x 10^5^ MC38 cells to the opposite flanks of mice was performed 3 months after the first rechallenge, and signs of tumor growth was followed for 60 days.

**Supplementary Fig. 7: Markers for different cell populations analyzed by single-cell RNA-seq, related to Figure 7.** Bubbleplot showing the average expression and percent positive cells for marker genes of each defined cell type. Cluster cell type assignments were determined using Seurat’s FindMarkers function with default parameters.

**Supplementary Fig. 8: Pro-VSV^IN^-IL-12 activates Th1 cells and inhibits Tregs in the tumor, related to Fig. 7.** **a,** UMAPs of CD4^+^ T cells colored by T conventional cell (Tconv) and T regulatory cell (Treg) clusters. **b,** Proportion of Treg and Tconv within each treatment group. **c,** Violin plot of *Ifng* expression in CD4^+^ Tconv cells within each treatment group. **d,** Violin plot of T-bet (*Tbx21*) expression in CD4^+^ Tconv cells within each treatment group. **e,** Proportion of T-bet^+^ Foxp3^+^ double positive Tregs within each treatment group. **f,** Violin plot of *Tbx21* expression in CD4^+^ Treg cells within each treatment group. **g,** Violin plot of CD103 (*Itgae*) expression in CD4^+^ Treg cells within each treatment group.

**Supplementary Fig. 9: Enhanced CD8^+^ T cell clonal expansion following Pro-VSV^IN^-IL-12 treatment, related to Fig. 7. a**, Bar graph of CD8^+^ clonal expansion and associated UMAPs for each treatment condition. Expanded and hyperexpanded CD8^+^ T cell clones were defined as clones which represented greater than 1% and less than 5% of total CD8^+^ T cells, and clones that represented greater than 5% of CD8^+^ T cells, respectively. **b**, Heatmap of CD8^+^ T cell associated markers between different clonal expansion groups.

**Supplementary Fig. 10:** **CD8^+^ T Cells are required for effective treatment by Pro-VSV^IN^-IL-12, related to Fig. 7. a-b:** Heatmap of gene signatures within Monocyte **(a)** and NK cell **(b)** clusters between different treatment groups. **c,** 5 x 10^5^ MC38 tumor cells were subcutaneously grafted to C57BL6/J mice. When tumors reached a volume of approximately 50 mm^3^, mice were treated i.v. with PBS or Pro-VSV^IN^-IL-12 every 3 days for 4 treatments. At the time of these injections, depleting antibodies against CD4, CD8, and NK1.1, or isotype control antibodies, were administrated by intraperitoneal injection. Tumor volumes for each treatment cohort were monitored over time, n = 9 - 10. **d,** C57BL6/J mice were treated intraperitoneally with isotype, anti-CD4, anti-CD8, or anti-NK1.1 antibodies, and spleens were harvested 3 days later and analyzed by flow cytometry to confirm the depletion of each immune population. Data is representative of findings in 3 animals per treatment group.

**Supplementary Fig. 11: Combined treatment with Pro-VSV^IN^-IL-12 and αPD-1 is well tolerated by mice, related to Figure 8.** Effect of treatment with **(a)** isotype, **(b)** Pro-VSV^IN^-IL-12 + isotype, **(c)** αPD-1, and **(d)** Pro-VSV^IN^-IL-12 + αPD-1 on the bodyweight of mice, n = 10 - 11.
